# Taxonomic Reference Libraries for Environmental Barcoding: A Best Practice Example from Diatom Research

**DOI:** 10.1371/journal.pone.0108793

**Published:** 2014-09-29

**Authors:** Jonas Zimmermann, Nelida Abarca, Neela Enk, Oliver Skibbe, Wolf-Henning Kusber, Regine Jahn

**Affiliations:** 1 Botanic Garden and Botanical Museum Berlin-Dahlem, Freie Universität Berlin, Berlin, Germany; 2 Justus-Liebig-Universität Giessen, AG Spezielle Botanik, Giessen, Hessen, Germany; 3 Larger than life - micro and nature photography, http://www.larger-than-life.de, Berlin Germany; University of Veterinary Medicine Hanover, Germany

## Abstract

DNA barcoding uses a short fragment of a DNA sequence to identify a taxon. After obtaining the target sequence it is compared to reference sequences stored in a database to assign an organism name to it. The quality of data in the reference database is the key to the success of the analysis. In the here presented study, multiple types of data have been combined and critically examined in order to create best practice guidelines for taxonomic reference libraries for environmental barcoding. 70 unialgal diatom strains from Berlin waters have been established and cultured to obtain morphological and molecular data. The strains were sequenced for 18S V4 rDNA (the pre-Barcode for protists) as well as *rbc*L data, and identified by microscopy. LM and for some strains also SEM pictures were taken and physical vouchers deposited at the BGBM. 37 freshwater taxa from 15 naviculoid diatom genera were identified. Four taxa from the genera *Amphora*, *Mayamaea*, *Planothidium* and *Stauroneis* are described here as new. Names, molecular, morphological and habitat data as well as additional images of living cells are also available electronically in the AlgaTerra Information System. All reference sequences (or reference barcodes) presented here are linked to voucher specimens in order to provide a complete chain of evidence back to the formal taxonomic literature.

## Introduction

Diatoms are unicellular and usually photoautotroph micro algae which are responsible for about 25% of global CO_2_ fixation [1]–[3] and contribute approximately 20% of the global net primary production [4].

Diatoms are important bioindicators for monitoring water quality because they are sensitive to changes in pollution, nutrient availability, acidity and salinity, e.g. [5], [6]. They are the most ubiquitous group within the microscopic algae as they occur in all types of water bodies and play an important part in benthic and planktonic biocoenoses [7]. They are routinely used as bioindicators within the EU Water Framework Directive (WFD) as well as in water quality monitoring worldwide [8]–[13].

Each diatom cell is encased in two siliceous shells (frustules) that are connected by girdle bands [1]–[3]. Current identification of diatoms is based on a morphological and mostly descriptive species concept (Zimmermann et al. subm.) and relies exclusively on micro-characters of the frustule such as size, symmetry, shape, and sculpture which can be seen by light microscopy [14]; more detailed analyses of the siliceous structures lead to more and more refined differentiation of species, which is possible through the development of higher resolution techniques, e.g. electron microscopy.

Identification via microscopy is challenging and time consuming, especially for routine use [15], and relies on individual taxonomic expertise. Therefore different taxonomists could arrive at different conclusions, depending i.a. on the taxonomic concept, species with limited diagnostic morphological features, cryptic species, available reference floras and quality of microscopes used by each individual researcher [15] as well as unavailability of adequate descriptions.

**Figure 1:**
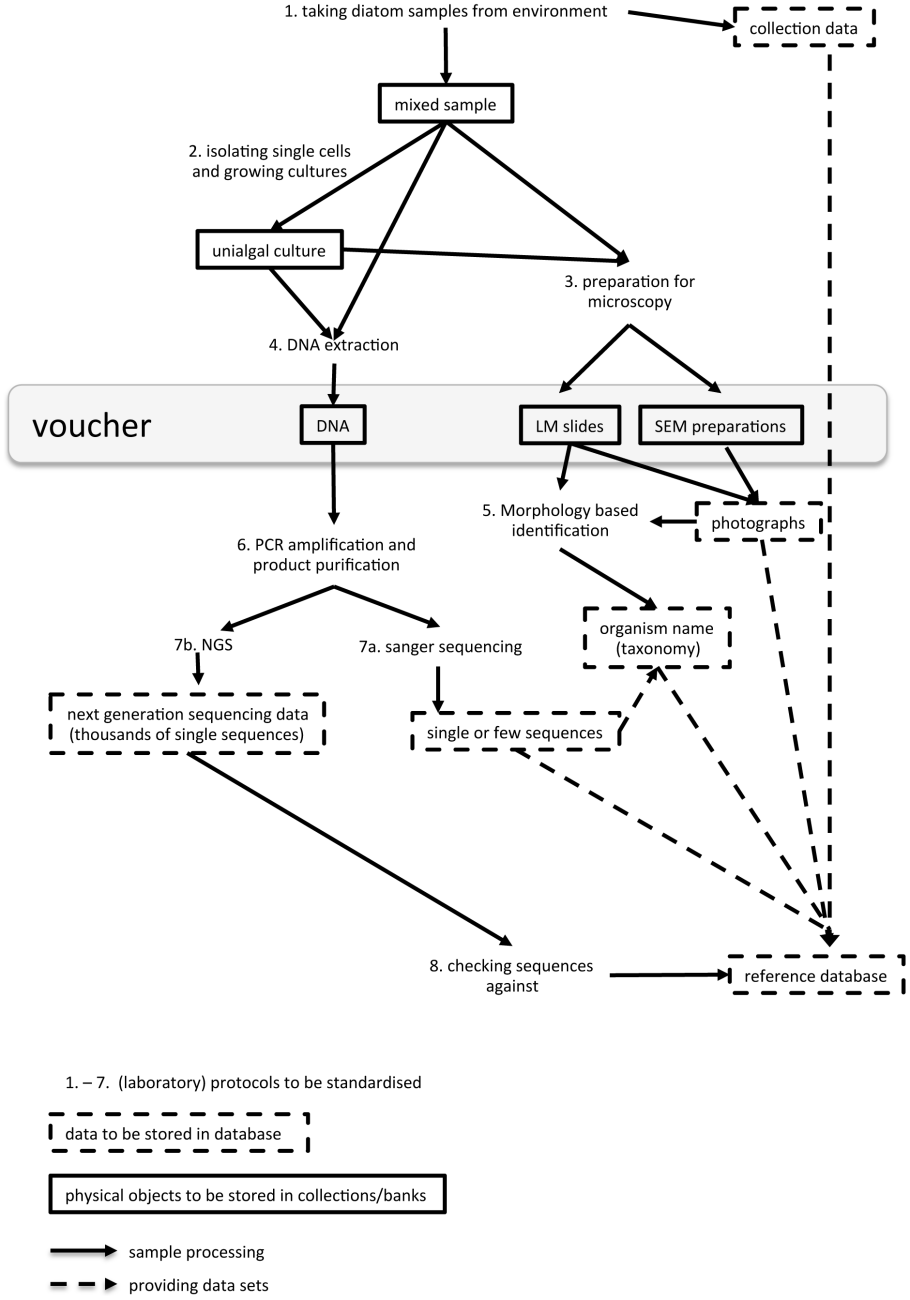
Schematic overview of sample processing and voucher as well as data production and deposition for environmental barcoding.

The application of molecular markers for taxon identification – DNA barcoding – is an emerging method which has the potential to be faster, universally applicable and generate reliable identification. Furthermore, as it uses DNA sequences for identification, it is independent of pre-existing morphological species concepts and can be linked to any taxonomic concept [16]. However, correct identification relies fundamentally on the quality of the reference library the DNA barcodes are checked against. DNA barcoding is based on the assumption that sequences of a certain marker locus exhibit enough variation between species to be discriminative for unambiguous species discovery [17], [18]. DNA barcoding is also a useful tool to access concealed diversity e.g. [19]–[25]. DNA barcoding in combination with next generation sequencing techniques also allows for the description of community compositions through the large numbers of sequences generated by this approach e.g. [26], [27]. A schematic overview on environmental DNA barcoding of diatoms and the establishment of a reference library is given in Fig. 1.

The requirement for reliable taxon identification by DNA barcode(s) is an unambiguous link between the genotype and the phenotype (or morphotype) to which the name of the species is attached. This means that a reference library consisting of taxon names belonging to specimens that have been identified by experts as well as providing descriptions together with barcode sequences, which were derived from well documented strains (e.g. voucher deposition, sampling localities and collectors, basic environmental data, high-resolution LM pictures, morphometrics, taxonomy and nomenclature, maps, literature and references to databases where this data is deposited) for every single species is necessary. For unicellular diatoms, clone cultures (strains) need to be established which offer enough material for sequencing as well as for identification by light and electron microscopy. Once established and linked to a taxonomic reference library, the DNA barcoding method could offer a time and cost efficient alternative/extension to microscopic identification for routine applications by limiting morphological taxonomy to critical groups which feature a distinct genetic aberration to known and identified organisms in the library.

Recently, the CBOL Protist Working Group [28] has designated the 18S V4 rDNA marker region as first or pre-barcode for Protist organisms. In this paper, we follow the 18S V4 protocols designed for diatoms by Zimmermann et al. [19], and present 70 strains for which this pre-barcode (18S V4) as well as a second widely used barcode, *rbc*L [20], [21], [29], has been generated. The reference library includes these two DNA barcodes, the respective taxon name, images, morphometric and geographic data as well as vouchers for further reference. Further data and additional images also of living cells are available electronically through the AlgaTerra Information System [30]. We demonstrate the benefits of a well documented reference library for DNA barcoding for identification, taxonomy, phylogeny, and further scientific analyses on an exemplary group. This paper focuses on naviculoid diatom strains from Berlin waters since its diatom flora has been well studied for almost two centuries by light microscopy [31] and a recent diatom flora is available for water quality assessments [32].

## Materials and Methods

### Sampling

**Figure 2:**
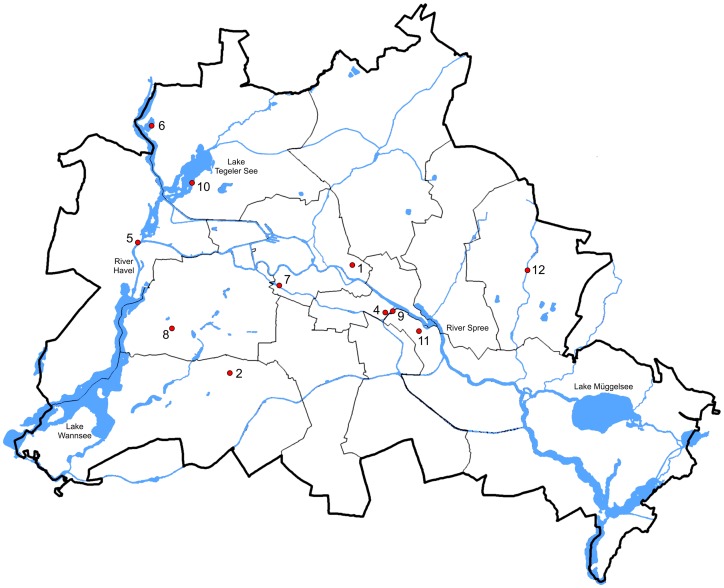
Map of the sampling localities in the Berlin region. For details to the numbered sampling sites see Table 1.

Benthic samples from which the 70 strains were established were collected at 11 sites in the catchment area of Berlin (Fig. 2); one additional sample was from the River Elbe, downstream of the Berlin Rivers Spree and Havel. Conductivity of Berlin water ranges mostly between 400 to 900 µS cm^−1^, pH is frequently 6,5 to 9 (80% respectively 88% of about 300 measurements of Berlin water samples, Kusber unpubl. data). For samples, sites, dates, collectors of the samples and isolators of the strains see Table 1. No specific permissions were required for the sampled locations/activities. The field studies did not involve endangered or protected species.

**Table 1:** List of Localities: Strain Numbers, Geo-Reference, Habitat, Ecology.

Locality	Geo references*	Date	Habitat	Ecology	Strains	collected and isolated
1. Berlin**	N 52.518611°	2005	Freshwater	E	Amph5	O. Skibbe
	E 13.408056°					
2. Dahlem, Berlin	N 52.460833°	19 Oct. 2004	Agricultural soil	T, E	D27_003, D27_006, D27_009 D28_001 D28_004b, D28_007b D29_003b, D29_009b, D30_003 D30_006b, D30_009	L. Buhr (coll.), O. Skibbe (isol.)
	E 13.296944°					
3. Elbe near Schnackenburg, Lower Saxony	N 53.039634°	26 Oct. 2009	River	T, E	ElCal01a, ElPin01	D. Borgwardt (coll.), O. Skibbe (isol.)
	E 11.564806°					
4. Görlitzer Park, Berlin	N 52.494850°	May 2006	Pond	P, E	PinnB	O. Skibbe
	E 13.443891°					
5. Havel at Spandau, Berlin	N 52.534512°	June 2006	River	T, E	Amph4	O. Skibbe
	E 13.204309°					
6. Heiligensee, Berlin	N 52.60394°	August 2011	Shallow lake	D, E	HSB02	O. Skibbe
	E 13.21499°					
7. Landwehrkanal, Berlin	N 52.510733°	11 June 2005	Canal	T, E	D36_003, D36_012, D36_020	W.-H. Kusber (coll.), J. Bansemer (isol.)
	E 13.338638°					
8. Ökowerk, Berlin	N 52.49179°	June 2006	Artificial moorland pool	P, A	PinnC	O. Skibbe
	E 13.23589°					
9. Spree at Kreuzberg, Berlin	N 52.49491°	March/April 2004	River	T, E	Amph1, Coco1, Pinn1	O. Skibbe
	E 13.44729°					
		22 March 2004			D03_030, D03_034, D03_063,	O. Skibbe (coll.), J. Bansemer (isol.)
					D03_074, D03_082, D03_093,	
					D03_139,	
		2007			D45_03,	O. Skibbe
		20 Sept. 2007			D54_02,	O. Skibbe
		October 2011			SpCo1	W. da Silva (coll.), O. Skibbe (isol.)
10. Tegeler See	N 52.57023°	September 2009	Lake	D, E.	TeAm01, TeNav01	R. Jahn (coll.), O. Skibbe (isol.)
	E 13.25691°					
11. Treptower Park, Berlin	N 52.48445°	May 2004	Pond	T, E	Navi1	O. Skibbe
	E 13.47148°					
12. Wuhle, Berlin	N 52.52079	21 April 2004	Small river	T, E	D06_006, D06_014, D06_023, Navi2,	O. Skibbe (coll.), J. Bansemer (isol.)
	E 13.57781°					
					D06_029, D06_036, D06_038,	
					D06_047, D06_059, D06_060,	
					D06_067, D06_069, D06_074,	
					D06_077, D06_083, D06_087,	
					D06_093, D06_095, D06_096,	
					D06_102, D06_106, D06_107,	
					D06_110, D06_113, D06_122,	
					D06_138, D06_139,	
		April 2004			Navi4, Stau1	O. Skibbe
		June 2004			Navi5, Pinn2	O. Skibbe

(Ecology: D = dimictic, P = polymictic, T = turbid, E = eutrophic, alkaline, A = acidic, all running waters are part of Elbe catchment area).

*Uncertainty = ±50 m,

** Uncertainty = ±22500 m.

### Cultivation

The diatom cells were isolated from environmental water samples observed under a stereo light microscope using capillary glass pipettes. The respective cell was then transferred to a 5 cm diameter plastic petri dish containing autoclaved habitat water and/or culture medium (WC [33], Chu [34], AlgaGrow, Plagron, Weert, Netherlands) of adequate salinity and pH. In order to remove unwanted particles, this treatment was repeated several times until microscopic inspection confirming that a culture derived from one cell, but not axenic had been established. The cultures were grown at a temperature between 18–22°C and a 12 h day/night cycle.

### Preparation of frustules

By the time of harvesting the cultures, one fraction was used for obtaining DNA (see below) and the other part was cleaned with H_2_0_2_ at 80°C and rinsed several times with H_2_0. A few drops of the resulting suspension of diatom frustules were dried on a cover slip and embedded as slides in Naphrax for study in LM or on stubs if for SEM. Vouchers of each strain were deposited in the Herbarium Berolinense (B) (see Table 2).

**Table 2:** List of materials from Berlin localities: names of 37 Taxa and 70 strains, voucher codes in the Herbarium Berolinense (B), DNA Bank voucher numbers in the Berlin-Dahlem Plant DNA Bank, INSDC accession numbers, picture numbers in this publication and morphometrics of each strain (n = number of evaluated valves).

Taxon	Strain	B Voucher*	DNA Bank no.	INSDC no. 18S V4 rRNA	INSDC no. *rbc*L	Figures in this paper**	Morphology length µm	Morphology breadth µm	Morphology striae/10 µm [punctae/10 µm]	n
*Achnanthidium saprophilum* (H.Kobayasi & Mayama) Round & Bukht.	D06_036	B400040822	DB 8634	KM084866	KM084941	3.1	12.8–13.7	3.2–3.9	27–31	8
*Amphora berolinensis* N.Abarca & R. Jahn sp. nov.	HSB02	B400040823 Holotype	DB 8635	KM084914	KM084982	4.1a–i	9.5 18.9	4.7–5.2	15	5
*Amphora ovalis* (Kütz.) Kütz.	Amph1	B400040825	DB 8637	KM084867	KM084926	-	22.9–28.5	7.5–9.2	13–14	7
	Amph4	B400040826	DB 8638	KM084868	KM084927	3.11	41.5–48.6	10.9–11.1	13	7
	Amph5	B400040827	DB 8639	KM084869	KM084928		33.7–37	7.8–9.4	11–13	4
	D45_003	B400040824	DB 8636	KM084910	KM084978	-	78.5–79.1	13.5–16.1	11–12	4
	TeAm01	B400040828	DB 8640	KM084924	KM084993		61–67.3	13–17.5	11–13	5
*Amphora pediculus* (Kütz.) Grunow	D03_074	B400040829	DB 8641	KM084872	KM084933	3.8	11.9–12.6	3.0–3.8	20–21	7
*Amphora* cf. *pediculus* (Kütz.) Grunow	D03_063	B400040830	DB 8642	KM084871	KM084932		9.9–13.3	3.2–4.1	19–21	7
	D03_082	B400040831	DB 8643	KM084873	KM084934	3.10	11.5–14.7	2.7–3.6	20–21	8
*Amphora* sp. aff. atomoides Levkov	D54_002	B400040832	DB 8644	KM084911	KM084979	3.9	10.2–12.4	4.6–5	16	6
*Caloneis amphisbaena* (Bory) Cleve	ElCal01a	B400040834	DB 8646	KM084912	KM084980		72–74	23.5–24.7	15–17	7
	Navi1	B400040833	DB 8645	KM084915	KM084983	3.19	72.2–73.4	25.7–26.9	15–16	3
*Caloneis silicula* (Ehrenberg) Cleve	D06_074	B400040835	DB 8647	KM084883	KM084948		12.5–13	7–7.7	19–20	10
*Cocconeis pediculus* Ehrenberg	Coco1	B400040687	DB 8209	FR873234	KM084929		22.5–29.1	18.2–21-8	19	3
	D36_020	B400040644 [Epitype]	DB 8208	FR873233	KM084977	3.12	16.6–40	13–26	14–22	50
	SpCo1	B400040808	DB 8648	KM084923	KM084991		23–23.7	16.5–17.6	18–20	3
*Cocconeis placentula* Ehrenberg	D36_012	B400040647 [Epitype]	DB 8211	FR873236	KM084976	3.13	14.7–33.7	9.7_22.8	18–26	50
*Craticula cuspidata* (Kütz.) D.G.Mann	Navi4	B400040836	DB 8649	KM084917	KM084985	3.23	133.3	27.5	13	**3**
*Craticula buderi* (Hust.) Lange-Bert.	D06_069	B400040837	DB 8650	KM084882	KM084947	3.25	27.5–28.1	6.0–7.1	19–20	4
*Eolimna minima* (Grunow) Lange-Bert.	D03_030	B400040692	DB 8219	FR873244	KM084930	3.15	7.3–7.8	3.4–3.8	28	5
*Eolimna sp.* (teratological valves)	D06_023	B400040880	DB 8651	KM084877	KM084939		11.9–12.4	3.8–4.0	28	2
*Gomphonema saprophilum* (Lange-Bert. & Reichardt) N.Abarca, R.Jahn, J. Zimmermann & Enke	D36_003	B400040919 [Epitype]	DB 8652	HG530017	HG530056	3.34	24.5–26	6.5–8	14–20	10
*Karayevia ploenensis* var. *gessneri* (Hust.) Bukt.	D03_034	B400040838	DB 8653	KM084870	KM084931	3.6	13.5–14.0	4.9–5.4	16–18	6
*Luticola sparsipunctata* Levkov, Metzeltin & Pavlov	D06_029	B400040839	DB 8654	KM084878	KM084940	3.7	16.4–26.7	5.5–8.4	16–18	15
*Mayamaea terrestris* N.Abarca et R.Jahn sp. nov.	D27_003	B400040840	DB 8655	KM084897	KM084963		7.1–8.0	3.0–4.3	24–26	19
	D27_006	B400040841	DB 8656	KM084898	KM084964		7.5–7.9	3.5–4.1	22–24	9
	D27_009	B400040864	DB 8657	KM084899	KM084965		7.7–8.8	3.4–4	20–22	6
	D28_001	B400040843	DB 8658	KM084900	KM084966		7.0–8.7	3.7–4.5	22–24	17
	D28_004b	B400040844	DB 8659	KM084902	KM084968		7.4–7.6	3.6–4.1	20–22	9
	D28_007b	B400040845	DB 8660	KM084903	KM084969		7.5–8.6	3.5–4.2	21–22	12
	D29_003b	B400040846	DB 8661	KM084905	KM084971		7.7–8.5	3.6–4.4	22	5
	D29_009b	B400040847 Holotype	DB 8662	KM084906	KM084972	4.2a–g	7.2–8	3.3–3.7	22.5–27	17
	D30_003	B400040848	DB 8663	KM084907	KM084973		7.2–8.3	4.2–4.4	24	10
	D30_006b	B400040849	DB 8664	KM084908	KM084974		7.4–8.2	3.5–3.7	22–23	6
	D30_009	B400040850	DB 8665	KM084909	KM084975		7.3–8.0	3.8–4.3	22	5
*Mayamaea permitis* (Hust.) Bruder & Medlin	D06_106	B400040851	DB 8666	KM084891	KM084956	3.17;3.18	6.6–8.2	3.6–4.4	no data	17
	D06_107	B400040697	DB 8224	FR873249	KM084957		7.2–8.5	3.6–3.85	22	10
*Navicula cryptotenella* Lange-Bert.	TeNav01	B400040852	DB 8667	KM084925	KM084994		17.5–20.5	4.6–5.7	15–16	10
*Navicula cryptocephala* Kütz.	D06_059	B400040700	DB 8226	FR873251	KM084944	3.26	29.0–30.4	5.8–6.6	15–16	8
	D06_067	B400040853	DB 8668	KM084881	KM084946		28.0–30.4	5.7–6.8	15–17	10
*Navicula gregaria* Donkin	D06_077	B400040855	DB 8670	KM084884	KM084949		20.6–24.8	5.7–6.7	19	2
	D06_096	B400040854	DB 8669	KM084889	KM084954		27.5–27.7	6.5–6.9	18–19	3
	D06_122	B400040856	DB 8671	KM084894	KM084960	3.24	24.2–25.0	5.9–6.2	17–18	6
*Navicula radiosa* Kütz.	D06_102	B400040857	DB 8672	KM084890	KM084955	3.22	59.5–59.9	10.3–10.9	11–12 [24]	4
*Navicula rhynchotella* Lange-Bert.	D06_083	B400040860	DB 8676	KM084885	KM084950		42.2–43.0	11.0–11.8	9–10 [24]	4
	D06_087	B400040861	DB 8675	KM084886	KM084951		46.9–47.0	12.2–12.6	8–9 [24]	4
	D06_093	B400040858	DB 8673	KM084887	KM084952	3.21	43.2–45.8	11.7–12.4	8–10 [24]	6
	D06_095	B400040859	DB 8674	KM084888	KM084953		44.2–44.3	11.3–11.5	9–10 [24]	3
*Navicula slesvicensis* Grunow	D06_038	B400040705	DB 8228	FR873253	KM084942	3.27	42.2–44.0	8.9–9.4	9–10 [24]	5
*Navicula tripunctata* (O.F.Müll.) Bory	D03_093	B400040706	DB 8230	FR873255	KM084935		32.5–36.3	7.6–8.0	10–11	6
	D03_139	B400040862	DB 8677	KM084874	KM084936	3.20	42.0–43.2	7.6–8.3	11–12	5
	Navi5	B400040863	DB 8678	KM084918	KM084986		28.5–31.3	7.5–8.0	11–12	7
*Pinnularia neomajor* Krammer	Pinn1	B400040865	DB 8679	KM084919	KM084987		185.1–206.2	27.0–28.5	6–7	4
	PinnB	B400040866	DB 8680	KM084921	KM084989	3.29	196.8–202.0	26.7–27.7	7	3
*Pinnularia viridiformis* Krammer	ElPin01	B400040870	DB 8682	KM084913	KM084981	3.32	69.5–77.6	15–17.2	8–9	8
	Pinn2	B400040867	DB 8681	KM084920	KM084988	3.31	78.1–84.9	17.2–17.9	8–9	2
*Pinnularia* sp.	Navi2	B400040868	DB 8683	KM084916	KM084984	3.33	31.1–35.6	10.8–11.3	10.5–11	8
*Pinnularia* sp.	PinnC	B400040869	DB 8684	KM084922	KM084990	3.30	91.7–93.4	17.8–18.4	6–7	3
*Planothidium caputium* J.Zimmermann, & R.Jahn sp. nov.	D06_014	B400040871 Holotype	DB 8687	KM084876	KM084938	4.3a–h	20.7–22.9	6.0–6.4	13–14	4
	D06_113	B400040875	DB 8688	KM084893	KM084959		20.3–21	5.5–6.2?	13–15	8
*Planothidium frequentissimum* (Lange-Bert.) Lange-Bert.	D06_138	B400040872	DB 8685	KM084895	KM084961		14.7–16.7	5.4–5.9	14–15	9
	D06_139	B400040873	DB 8686	KM084896	KM084962	3.2;3.3	14–6-16	5.6–6.2	14–16	4
*Planothidium lanceolatum* (Bréb. ex Kütz.) Lange-Bert.	D06_047	B400040874	DB 8689	KM084879	KM084943	3.4;3.5	10.1–12.5	4.6–5.1	13–14	20
*Sellaphora pupula* (Kütz.) Mereschk.	D06_060	B400040877	DB 8690	KM084880	KM084945	3.16	17.7–18.2	6.7–7.0	21–24	5
	D06_110	B400040878	DB 8691	KM084892	KM084958		17.8–18.1	6.7–7.0	21–22	7
*Sellaphora seminulum* (Grunow) D.G.Mann	D06_006	B400040879	DB 8692	KM084875	KM084937	3.14	11.1–18.1	3.8–4.8	20–22	9
*Stauroneis phoenicenteron* (Nitzsch) Ehrenb.	Stau1	B400040715	DB 8239	FR873264	KM084992	3.28	161.2–164.1	31.2–31.6	13–14	3
*Stauroneis schmidiae* R.Jahn & N.Abarca sp. nov.	D28_002	B400040883	DB 8693	KM084901	KM084967		25.2–27.4	5.5–6	17–19	3
	D28_008	B400040882 Holotype	DB 8694	KM084904	KM084970	4.4a–h	27–28.2	5.5–6	15–18	7

INSDC accession numbers starting with KM are newly published here, those starting with FR have been published in Zimmermann et al. (2011) [19] and numbers starting with HG in Abarca et al. (2014) [39].

* URL for AlgaTerra http://herbarium.bgbm.org/object/plus the corresponding B Voucher.

**pictures of all strains are available online by AlgaTerra [30].

### Light and electron microscopy

The LM pictures were acquired with a Zeiss Axio Imager.M2 with an implemented AxioCam HRc (Zeiss, Oberkochen, Germany). SEM pictures were produced with Philips SEM 515 operating at 30 KV (Philips, Eindhoven, The Netherlands), and Hitachi 8010 Field Emission Electron Microscope (Hitachi, Tokyo, Japan).

### Identification

The taxa were identified with Hofmann et al. [32], Krammer & Lange-Bertalot (1997) [35], Ettl & Gärtner (2013) [36], Lange-Bertalot (2001), Levkov et al. (2009) [37], Levkov et al. (2014) [38] as well as particular papers (vide infra) for selected species. For strain numbers, taxon names, voucher codes in the Herbarium Berolinense (B), EMBL Accession Numbers, images, and morphometric data for all strains see Table 2.

### DNA isolation

The harvested cultures were transferred to 1.5 ml tubes. DNA was isolated using Dynal DynaBeads (Invitrogen Corporation; Carlsbad, CA, USA), NucleoSpin Plant II Mini Kit (Machery and Nagel, Düren, Germany) or Qiagen Dneasy Plant Mini Kit (Qiagen Inc.; Valencia, CA) following the respective product instructions. DNA concentrations were checked using gel electrophoresis (1.5% agarose gel) and Nanodrop (PeqLab Biotechnology LLC; Erlangen, Germany). DNA samples were stored at −20°C until further use. DNA material was deposited in the Berlin collection of the DNA bank network [39].

### PCR amplification

The V4 region of the 18S locus was amplified in all strains with the primer pair M13F-D512 for 18S/M13F-D978rev 18S [19]. The *rbc*L locus was amplified in two overlapping parts using two different primer pairs; Diat-rbcL-F and Diat-rbcL-iR as well as Diat-rbcL-iF and Diat-rbcL-R [40] for all strains. The polymerase chain reaction (PCR) for the V4 region was conducted after Zimmermann et al. (2011) [19] and for *rbc*L carried out after Abarca et al. (2014) [40]. PCR products were visualised in a 1.5% agarose gel and cleaned with MSB Spin PCRapace (Invitek LLC; Berlin, Germany) following standard procedure. DNA content was measured using Nanodrop (PeqLab Biotechnology). The samples were normalised to a total DNA content >100 ng/µl using Nanodrop (PeqLab Biotechnology) for further sequencing.

### Sequencing

The Sanger sequencing was conducted by Starseq (GENterprise LLC; Mainz, Germany). As sequencing primers the M13 tails [19], [41] were used for the V4 region, following [42]. The sequences were edited in PhyDE [43] aligned using MUSCLE [44], and alignments were manually improved in PhyDE [43].

### Molecular analysis

The aligned sequences were compared to each other calculating uncorrected p distances in PAUP [45]. Then they were blasted against existing INSDC entries for the respective taxa (accessed July 2013). All INSDC accessions with references are given in Appendix S1. Base pair differences were counted in overlapping parts of the sequences in Mega 5 [46]. Results are summarised in Table 3.

**Table 3:** Sequence comparison of the here presented strains with corresponding accessions from INSDC databases.

Taxon	BGBM Strain	Accession INSDC (Strain)			
		18S V4	bp diff.	rbcL	bp diff.
*Achnanthidium saprophilum* (H.Kobayasi & Mayama) Round & Bukht.	D06_036	*Achnanthidium minutissima*			
		FR873231 (D05_008)^1^	15		
		AM502032 (AT-196Gel02)^1^	15	AM710499 (AT-196Gel02)^1^	21
		AJ866992 (AMIN)^1^	15		
*Amphora berolinensis* Abarca & R.Jahn	HSB02	*Amphora copulata* 2 (Kütz.)			
		Schoeman & R.E.M. Archibald			
		AM501959 (AT-117.10)	5	AM710425 (AT-117.10)	35
				JN162763 (IKCCMP0165)	42
*Amphora ovalis* (Kütz.) Kütz.	D45_003	-	-	-	-
	Amph1	-	-	-	-
	Amph4	-	-	-	-
	Amph5	-	-	-	-
	TeAm01	-	-	-	-
*Amphora pediculus* (Kütz.) Grunow	D03_074	AM501960 (AT-117.11)	3	AM710426 (AT-117.11)	14
				HQ912403 (L1030)	7
*Amphora* cf. *pediculus* (Kütz.) Grunow	D03_063	AM501960 (AT-117.11)	3	AM710426 (AT-117.11)	13
				HQ912403 (L1030)	2
	D03_082	AM501960 (AT-117.11)	3	AM710426 (AT-117.11)	14
				HQ912403 (L1030)	3
*Amphora* sp. aff. *atomoides* Levkov	D54_002	-	-	-	-
*Caloneis amphisbaena* (Bory) Cleve	Navi1	AM501954 (AT-177.07)	1	AM710507 (AT-177.07)	0
	ElCal01a	AM501954 (AT-177.07)	1	AM710507 (AT-177.07)	0
*Caloneis silicula* (Ehrenberg) Cleve	D06_074	JN418593 (Cal890TM)	0	JN418663 (Cal890TM)	0
*Cocconeis pediculus* Ehrenberg	D36_020	AM502010 (AT-212.07)	0	AM710477 (AT-212.07)	0
		FR873235 (LuCoc03)	0		
	Coco1	AM502010 (AT-212.07)	1	AM710477 (AT-212.07)	0
		FR873235 (LuCoc03)	1		
	SpCo1	AM502010 (AT-212.07)	2	AM710477 (AT-212.07)	0
		FR873235 (LuCoc03)	2		
*Cocconeis placentula* Ehrenberg	D36_012^1^	FR873239 (WiCoc01)	20		
		FR873237 (D17_011)	24		
		KC736616 (TCC501)	16	KC736591 (TCC501)	14
		HQ912592 (UTEXFD23)	23	HQ912456 (UTEXFD23)	40
		AM502013 (AT-212.Gel11)	19	AM710480 (AT-212.Gel11)	24
*Craticula cuspidata* (Kütz.) D.G.Mann	Navi4	HQ912581 (UTEX.FD35)	28	HQ912445 (UTEX.FD35)	10
*Craticula buderi* (Hust.) Lange-Bert.	D06_069	-	-	-	-
*Eolimna minima* (Grunow) Lange-Bert	D03_030	AM501962 (AT-70Gel18)	9	AM710427 (AT-70Gel18)	18
		AJ243063 (SNA15)	1		
		HM449712^3^	5		
*Eolimna* sp. (teratological valves)	D06_023	-	-	-	-
*Gomphonema saprophilum* Abarca et al.	D36_003	-	-	-	-
*Karayevia ploenensis* var. *gessneri* (Hust.) Bukt.	D03_034	-	-	-	-
*Luticola sparsipunctata* Levkov, Metzeltin & Pavlov	D06_029	-	-	-	-
*Mayamaea terrestris* Abarca & R. Jahn	D27_003	*Mayamaea atomus* (Kütz.) Lange-Bert var. atomus			
		AM501968 (AT-115Gel07)	5	AM710434 (AT-115Gel07)	25
				AM710510 (AT-199Gel01)	69
	D27_006	AM501968 (AT-115Gel07)	5	AM710434 (AT-115Gel07)	25
				AM710510 (AT-199Gel01)	69
	D27_009	AM501968 (AT-115Gel07)	5	AM710434 (AT-115Gel07)	25
				AM710510 (AT-199Gel01)	69
	D28_001	AM501968 (AT-115Gel07)	5	AM710434 (AT-115Gel07)	25
				AM710510 (AT-199Gel01)	69
	D28_004b	AM501968 (AT-115Gel07)	5	AM710434 (AT-115Gel07)	25
				AM710510 (AT-199Gel01)	69
	D28_007b	AM501968 (AT-115Gel07)	5	AM710434 (AT-115Gel07)	25
				AM710510 (AT-199Gel01)	69
	D29_003b	AM501968 (AT-115Gel07)	5	AM710434 (AT-115Gel07)	25
				AM710510 (AT-199Gel01)	69
	D29_009b	AM501968 (AT-115Gel07)	5	AM710434 (AT-115Gel07) AM710510 (AT-199Gel01)	25, 69
					
	D30_003	AM501968 (AT-115Gel07)	5	AM710434 (AT-115Gel07)	25
				AM710510 (AT-199Gel01)	69
	D30_006b	AM501968 (AT-115Gel07)	5	AM710434 (AT-115Gel07)	25
				AM710510 (AT-199Gel01)	69
	D30_009	AM501968 (AT-115Gel07)	5	AM710434 (AT-115Gel07)	25
				AM710510 (AT-199Gel01)	69
*Mayamaea permitis* (Hust.) Abarca & R.Jahn comb. nov.	D06_107	AM501969 (AT-101Gel04)	0	AM710435 (AT-101Gel04)	27
		JN418600 (Wes2f)	42	JN418670 (Wes2f)	34
	D06_106	AM501969 (AT-101Gel04)	0	AM710435 (AT-101Gel04)	27
		JN418600 (Wes2f)	35	JN418670 (Wes2f)	34
*Navicula cryptocephala* Kütz	D06_059	KC736631 (TCC515)	4	KC736601 (TCC515)	8
		HQ912603 (UTEX FD109)	0	HQ912467 (UTEX FD109)	0
				HQ337543 (CCMP2519)	19
		AM501996 (AT-176Gel05)	0	AM710463 (AT-176Gel05)	0
		AM501973 (AT-114Gel08c)	0	AM710439 (AT-114Gel08c)	0
	D06_067	KC736631 (TCC515)	4	KC736601 (TCC515)	13
		HQ912603 (UTEX FD109)	0	HQ912467 (UTEX FD109)	3
				HQ337543 (CCMP2519)	24
		AM501996 (AT-176Gel05)	0	AM710463 (AT-176Gel05)	3
		AM501973 (AT-114Gel08c)	0	AM710439 (AT-114Gel08c)	3
*Navicula cryptotenella* Lange-Bert.	TeNav01	AM502011 (AT-212Gel01)	1	AM710478 (AT-212Gel01)	0
		AM502029 (AT-202Gel03)	0	AM710496 (AT-202Gel03)	0
		AM502015 (AT-210Gel05)	0	AM710482 (AT-210Gel05)	0
*Navicula gregaria* Donkin	D06_096	FR873252 (D08_002)	1		
		HM805037 (BA102)	1		
		AM501974 (AT-117Gel05)	1	AM710440 (AT-117Gel05)	1
	D06_077	FR873252 (D08_002)	0		
		HM805037 (BA102)	0		
		AM501974 (AT-117Gel05)	0	AM710440 (AT-117Gel05)	1
	D06_122	FR873252 (D08_002)	0		
		HM805037 (BA102)	0		
		AM501974 (AT-117Gel05)	0	AM710440 (AT-117Gel05)	1
*Navicula radiosa* Kütz.	D06_102	AM502034 (AT-205.02b)	0	AM710501 (AT-205.02b)	0
		AM502027 (AT-200.04)	0	AM710494 (AT-200.04)	0
		AM501972 (AT-114Gel06)	0	AM710438 (AT-114Gel06)	0
*Navicula rhynchotella* Lange-Bert.	D06_093	-	-	-	-
	D06_095	-	-	-	-
	D06_087	-	-	-	-
	D06_083	-	-	-	-
*Navicula slesvicensis* Grunow	D06_038	-	-	-	-
*Navicula tripunctata* (O.F.Müll.) Bory	D03_093	AM502028 (AT-202.01)	0	AM710495 (AT-202.01)	0
	D03_139	AM502028 (AT-202.01)	0	AM710495 (AT-202.01)	0
	Navi5	AM502028 (AT-202.01)	0	AM710495 (AT-202.01)	0
*Pinnularia neomajor* Krammer	Pinn1	JN418585 (Corsea2)	0	JN418655 (Corsea2)	0
		JN418571 (Tor1a)	31	JN418641 (Tor1a)	15
	PinnB	JN418585 (Corsea2)	0	JN418655 (Corsea2)	0, 15
		JN418571 (Tor1a)	31	JN418641 (Tor1a)	
*Pinnularia viridiformis* Krammer	Pinn2	JN418589 (Pin870MG)	22	JN418659 (Pin870MG)	19
		JN418574 (Enc2a)	26	JN418644 (Enc2a)	24
		AM501985 (AT-70.10)	9	AM710451 (AT-70.10)	5
		AM743108 (L1716)	97		
	ElPin01	JN418589 (Pin870MG)	22	JN418659 (Pin870MG)	19
		JN418574 (Enc2a)	26	JN418644 (Enc2a)	24
		AM501985 (AT-70.10)	9	AM710451 (AT-70.10)	5
		AM743108 (L1716)	97		
*Pinnularia* sp.	Navi2	-	-	-	-
*Pinnularia* sp.	PinnC	-	-	-	-
*Planothidium frequentissimum* (Lange-Bert.) Lange-Bert.	D06_138	-	-	-	-
	D06_139	-	-	-	-
*Planothidium caputium* R.Jahn & Abarca sp. nov.	D06_014	-	-	-	-
	D06_113	-	-	-	-
*Planothidium lanceolatum* (Bréb. ex Kütz.) Lange-Bert.	D06_047	AJ535189 (L1249)	2	JQ610173 (LCR-S2-1-1)	17
*Sellaphora pupula* (Kütz.) Mereschk.	D06_060	EF151973 (Bel2)	1	EF143266 (Bel2)	0
		EF151983 (Aus4)	1	EF143317 (Aus4)	15
	D06_110	EF151973 (Bel2)	1	EF143266 (Bel2)	0
		EF151983 (Aus4)	1	EF143317 (Aus4)	15
*Sellaphora seminulum* (Grunow) D.G.Mann	D06_006	EF151967 (TM37)	0	EF143280 (TM37)	32
		KC736642 (TCC461)	22	KC736613 (TCC461)	16
*Stauroneis phoenicenteron* (Nitzsch.) Ehrenb.	Stau1	AM502031 (AT-182.07)	0	AM710498 (AT-182.07)	0
		AM501987 (AT-117.04)	2	AM710453 (AT-117.04)	0
*Stauroneis schmidiae* R.Jahn & Abarca sp. nov.	D28_002	-	-	-	-
	D28_008	-	-	-	-

Basepair differences (bp diff.) for each taxon and strain number specified for both markers 18S V4 and *rbc*L. – denotes missing representative for taxon in INSDC databases (accessed July 2013).

1 *Achnanthidium minutissimum* (Kützing) Czarnecki.

2 new name for the taxon formerly identified as *Amphora libyca* Ehrenberg.

3 as *Navicula minima*.

### Tree building

To identify molecular relations between the here presented strains, trees were calculated with Mega 5 using the Neighbour Joining algorithm with gamma distributed rates among sites followed by a statistical test of the tree topologies with 10 000 bootstrap replications. Trees for the individual alignments of 18S V4 and *rbc*L sets as well as a concatenated dataset were calculated.

Furthermore, we created 18S V4 as well as *rbc*L datasets including INSDC sequences for the genera *Amphora*, *Mayamaea*, *Planothidium* and *Stauroneis* to exemplarily test the taxonomic consistency of available sequences as well as the placement of our new taxa. Each of these eight datasets was analysed under the aforementioned conditions.

### Nomenclature

The electronic version of this article in Portable Document Format (PDF) in a work with an ISSN or ISBN will represent a published work according to the International Code of Nomenclature for algae, fungi, and plants, and hence the new names contained in the electronic publication of a PLOS ONE article are effectively published under that Code from the electronic edition alone, so there is no longer any need to provide printed copies. The online version of this work is archived and available from the following digital repositories: PubMed Central, LOCKSS. http://edocs.fu-berlin.de/docs/content/below/index.xml.

## Results

### Morphological analyses

**Figure 3:**
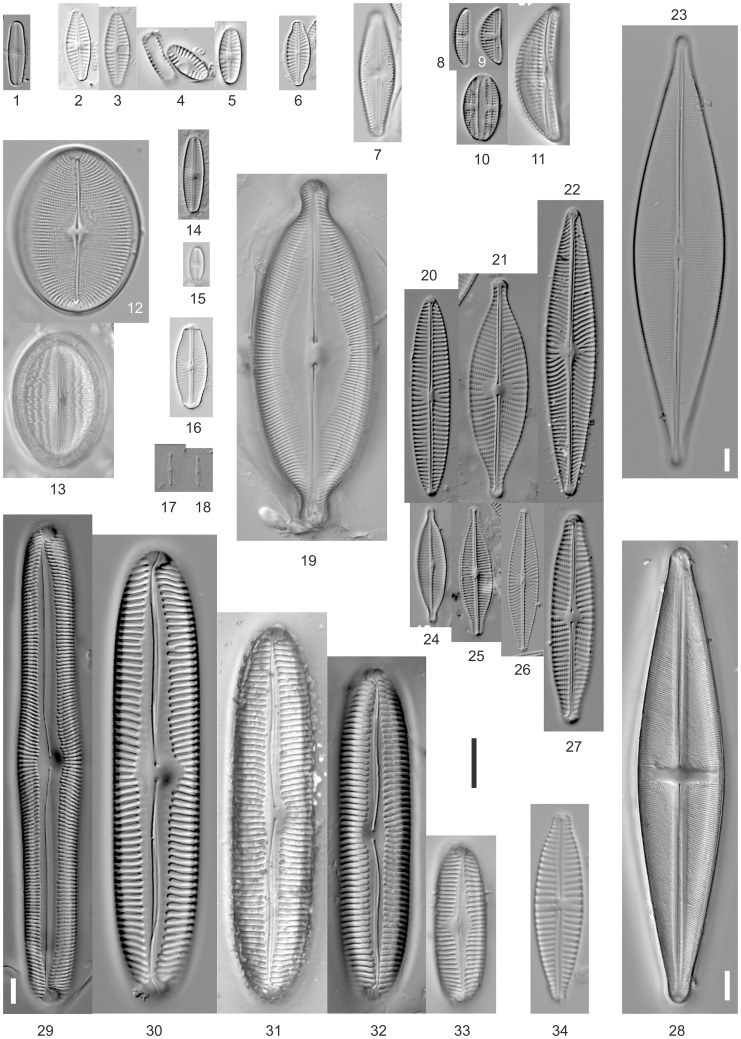
LM photos of individual valves from strains. Fig. 3.1. *Achnanthidium saprophilum* (H.Kobayasi & Mayama) Round & Bukht., Strain D06_036. Fig. 3.2.–3. *Planothidium frequentissimum* (Lange-Bert.) Lange-Bert., Strain D06_139. Fig. 3.4.–5. *Planothidium lanceolatum* (Bréb. ex Kütz.) Lange-Bert., Strain D06_047. Fig. 3.6. *Karayevia ploenensis* var. *gessneri* (Hust.) Bukt., Strain D03_034. Fig. 3.7. *Luticola sparsipunctata* Levkov, Metzeltin & Pavlov, Strain D06_029. Fig. 3.8. *Amphora pediculus* (Kütz.) Grunow, Strain D03_074. Fig. 3.9. *Amphora* sp. aff. *atomoides* Levkov, strain D54_002. Fig. 3.10. *Amphora* cf. *pediculus* (Kütz.) Grunow, Strain D03_082. Fig. 3.11. *Amphora ovalis* (Kütz.) Kütz., Strain Amph4. Fig. 3.12. *Cocconeis pediculus* Ehrenberg, Epitype-Strain D36_020. Fig. 3.13. *Cocconeis placentula* Ehrenberg, Epitype-Strain D36_012. Fig. 3.14. *Sellaphora seminulum* (Grunow) D.G.Mann, Strain D06_006. Fig. 3.15. *Eolimna minima* (Grunow) Lange-Bert., Strain D03_030. Fig. 3.16. *Sellaphora pupula* (Kütz.) Mereschk., Strain D06_060. Fig. 3.17.–18. *Mayamaea permitis* (Hust.) Bruder & Medlin, Strain D06_106. Fig. 3.19. *Caloneis amphisbaena* (Bory) Cleve, Strain Navi1. Fig. 3.20. *Navicula tripunctata* (O.F.Müll.) Bory, Strain D03_139. Fig. 3.21. *Navicula rhynchotella* Lange-Bert., Strain D06_093. Fig. 3.22. *Navicula radiosa* Kütz., Strain D06_102. Fig. 3.23. *Craticula cuspidata* (Kütz.) D.G.Mann, Strain Navi4. Fig. 3.24. *Navicula gregaria* Donkin, Strain D06_122. Fig. 3.25. *Craticula buderi* (Hust.) Lange-Bert., Strain D06_069. Fig. 3.26. *Navicula cryptocephala* Kütz., Strain D06_059. Fig. 3.27. *Navicula slesvicensis* Grunow, Strain D06_038. Fig. 3.28. *Stauroneis phoenicenteron* (Nitzsch) Ehrenb., Strain Stau1. Fig. 3.29. *Pinnularia neomajor* Krammer, Strain PinnB. Fig. 3.30. *Pinnularia* sp., Strain PinnC. Fig. 3.31. *Pinnularia viridiformis* Krammer, Strain Pinn2. Fig. 3.32. *Pinnularia viridiformis* Krammer, Strain ElPin01. Fig. 3.33. *Pinnularia* sp., Strain Navi2. Fig. 3.34. *Gomphonema saprophilum* (Lange-Bert. & Reichardt) N.Abarca, R.Jahn, J. Zimmermann & Enke, Strain D36_003. Scale bar represents 10 µm.

**Figure 4:**
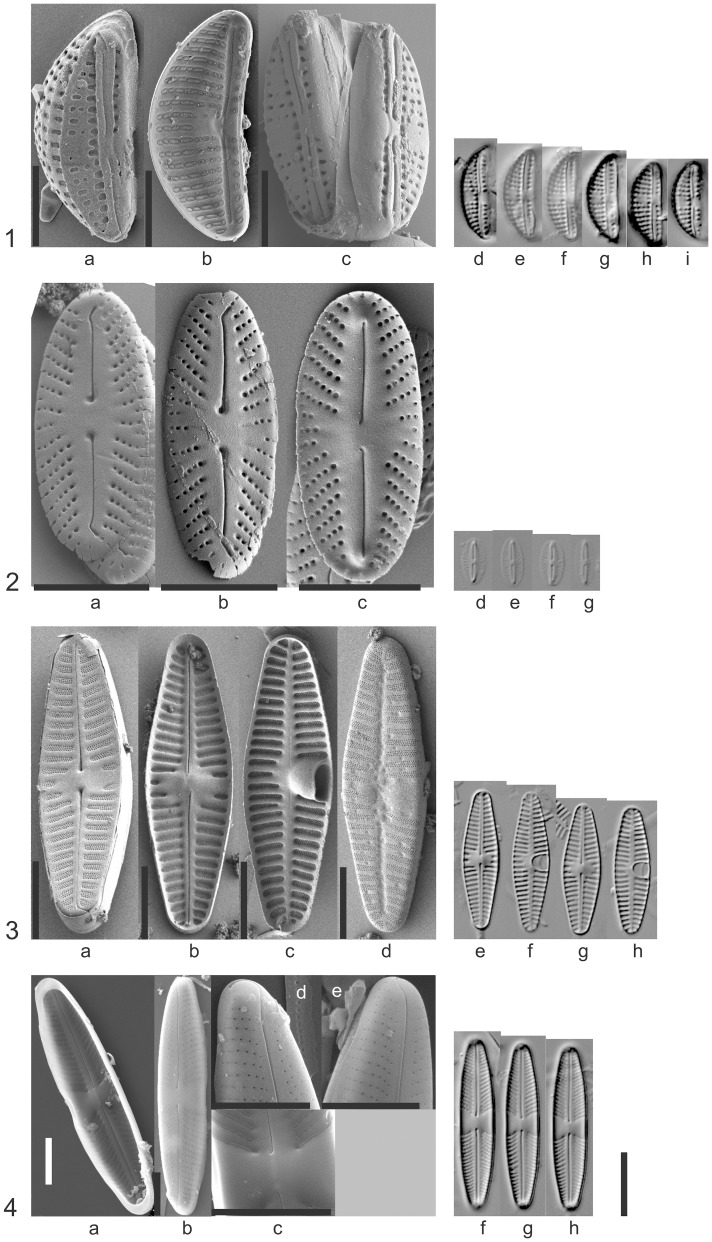
SEM and LM photos of the newly described species. Figs. 4.1a–i. *Amphora berolinensis* N.Abarca & R. Jahn sp. nov., Strain HSB02; Fig. 4.1d–i. Holotype B 40 0040823. Figs. 4.2a–g. *Mayamaea terrestris* N.Abarca et R.Jahn sp. nov., Strain D29_009b; Fig. 4.2d–g. Holotype B 40 0040847. Figs. 4.3a–h. *Planothidium caputium* J.Zimmermann & R.Jahn sp. nov., Strain D06_014; Fig. 4.3e–h Holotype B 40 0040871. Fig. 4.4a–h. *Stauroneis schmidiae* R.Jahn & N.Abarca sp. nov., Strain D28_008; Fig. 4.4f–h. Holotype B 40 0040882.

The morphological identification of the 70 strains resulted in 37 taxa (see Table 2 and Figs. 3 and 4). 21 taxa were identified by only one strain but 10 taxa were represented by two strains, three taxa by three strains, one taxon by four strains, one taxon by five strains and one taxon by 11 strains.

### DNA sequence analyses

PCR and sequencing success for 18S V4 and *rbc*L was 100% for all strains, resulting in 140 reference sequences for 70 strains. We established 129 novel sequences (INSDC accession numbers KM084866-KM084994) and an additional 11 sequences that had been previously published in Abarca et al. [40] and Zimmermann et al. [19].

There was little molecular variation within the here generated sequence data – only up to 0.5% in 18S V4 (representing 2 bp) and 0.3% in *rbc*L (corresponding to 3 bp) – between the different strains representing one taxon (Appendix S2). The highest in-taxon variation was found in e.g. *Mayamaea terrestris* 0.53% (18S V4), respectively *Navicula cryptocephala* e.g. 0.33% (*rbc*L). The uncorrected p distances for all genera and sequences are given in Appendix S2.

**Figure 5:**
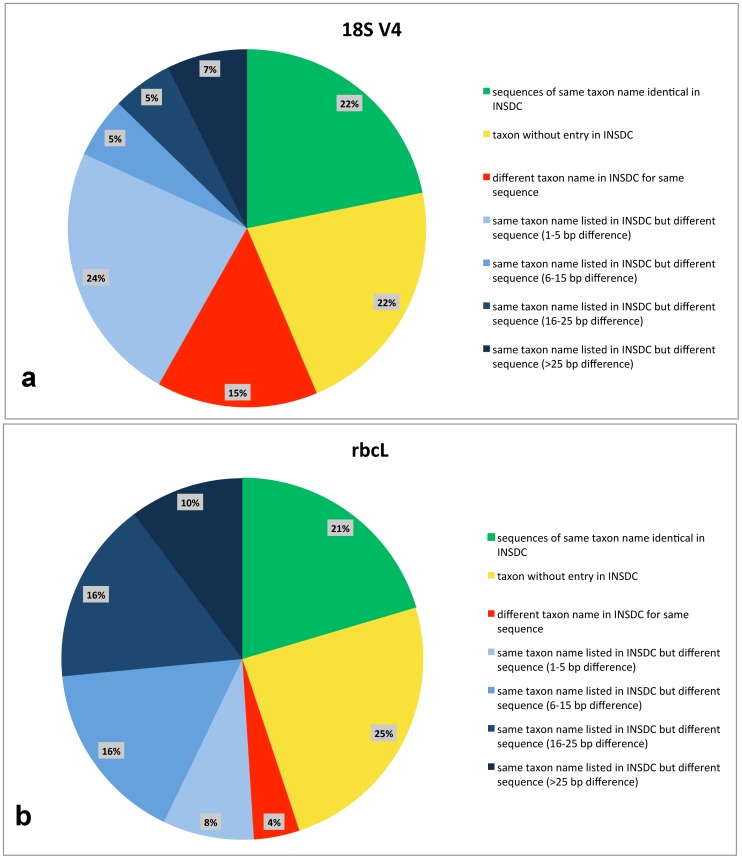
Chart giving classes of base pair (bp) differences for both markers (18S V4, *rbc*L) between here presented molecular data and corresponding data from INSDC databases. Inferred from data in Table 3.

The results from sequence comparison with sequences published in the databases of the International Nucleotide Sequence Database Collaboration (INSDC, includes GenBank, EMBL and DDBJ) are shown in Table 3 and summarised in Fig. 5a, 5b. In the case of 18S V4, 22% of our taxa had entries with identical sequences in the INSDC whereas for *rbc*L this number was 21% (Fig. 5b). This was the case e.g. for *Caloneis silicula* and *Navicula cryptotenella* (Table 3). 22% (18S V4, Fig. 5a) respectively 25% (*rbc*L, Fig. 5b) of our taxa had no entry in the INSDC databases, e.g. *Amphora ovalis* and *Luticola sparsipunctata* (Table 3). For 15% of our taxa an identical 18S V4 sequence (Fig. 5a) with a different taxon name was found in the INSDC databases (e.g. *Gomphonema parvulum*); the number was considerably lower in *rbc*L with only 4% (Fig. 5b). The remaining taxa of which many showed sequence dissimilarities of over 15 bp were 41% for 18S V4 (Fig. 5a) and 50% for *rbc*L (Fig. 5b). The highest difference was found for *Pinnularia viridiformis* with 97 bp in 18S V4 (Table 3).

**Figure 6:**
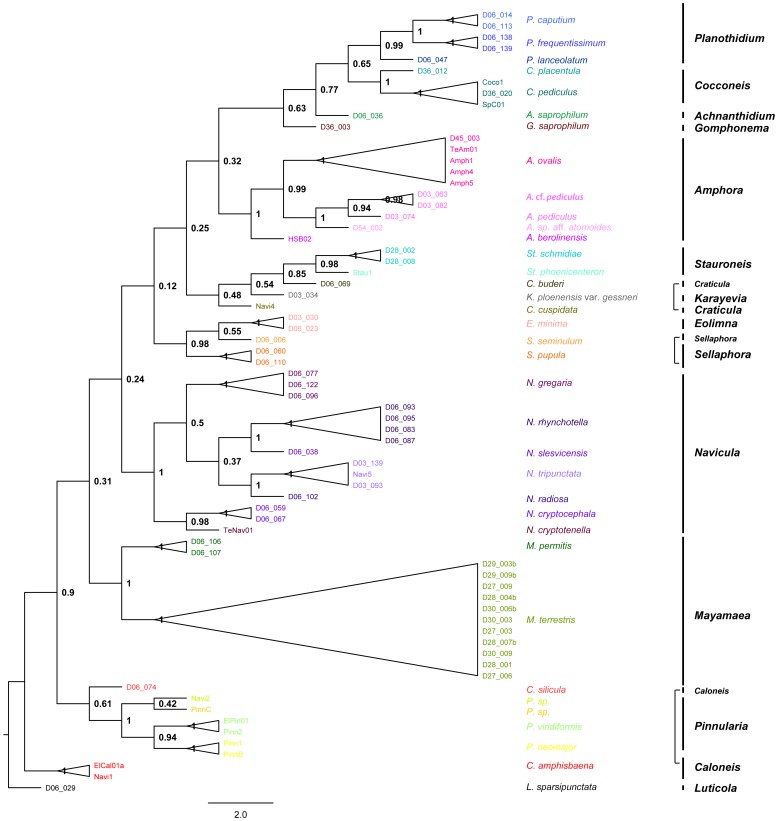
Neighbour Joining Tree (10 000 bootstrap replicates) derived from concatenated dataset (18S V4, *rbc*L) including all sequences from this study. All bootstrap support values given above branches.

The tree derived from the concatenated data set and calculated by the Neighbour Joining (NJ) algorithm, including only the here presented strains, is shown in Fig. 6; the trees of the individual analysis of both markers are given in the Appendix S2. The molecular clades are congruent between 18S V4 and *rbc*L, the tree topology is partly differing between both markers (Appendix S3, S4); however, the conflicting nodes have bootstrap values below 0.85 and are therefore neglected.

In the tree derived from the combined dataset, the sampled genera are monophyletic and well supported (>0.98 bootstrap support BS, Fig. 6), except for *Caloneis*, *Craticula* and *Sellaphora*.

*Craticula buderi* falls into a clade with the genera *Stauroneis* and *Karayevia* (0.48 BS; Fig. 6). *Sellaphora* falls into one group with *Eolimna* (0.98 BS; Fig. 6). The genus *Caloneis* is found in two distinct clades: *Caloneis silicula* is clustering with *Pinnularia* (0.61 BS; Fig. 6), *Caloneis amphisbaena* forms an independent clade on its own (1.00 BS; Fig. 6). The deeper bifurcations representing the relationship between the genera are generally not well supported by bootstrap values. All 37 subgeneric taxa included in this study are monophyletic (Fig. 6).

**Figure 7:**
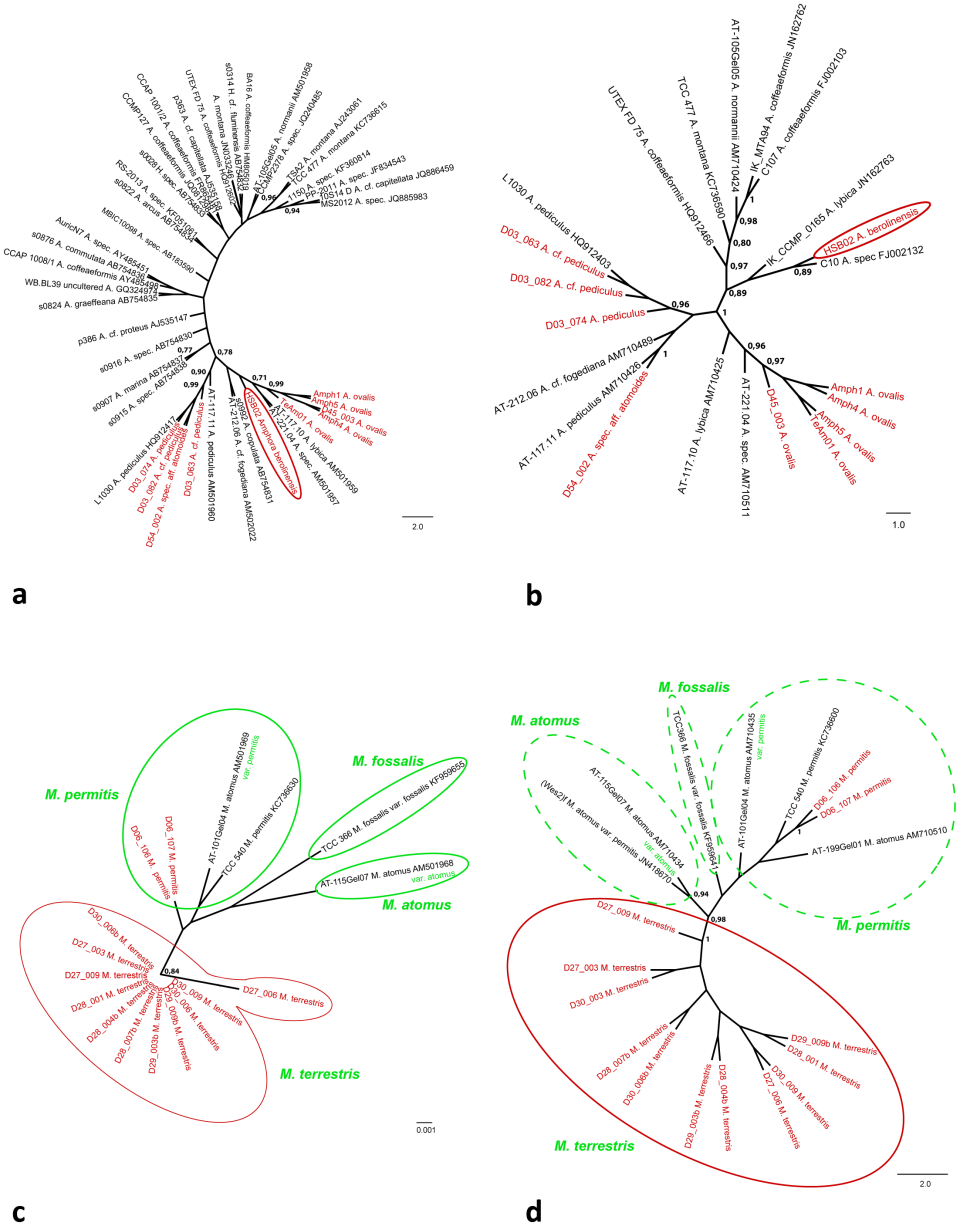
Neighbour Joining Tree (10 000 bootstrap replicates) including all sequences available from the INSDC databases for genus *Amphora* (a) 18S V4, (b) *rbc*L as well as *Mayamaea* (c) 18S V4, (d) *rbc*L. Bootstrap support values >0.75 given at nodes. Red indicates data from new species, green information and conlusions derived from data in the AlgaTerra Information System [30].

The trees for the genus *Amphora* including all available data from INSDC databases (this includes also accessions from the genus *Halamphora*) are shown in Fig. 7a (18S V4) and Fig. 7b (*rbc*L). The *Amphora ovalis* strains (Amph1, Amph4, Amph5, D45_003 and TeAm01) form a monophyletic clade, that is well supported in both 18S (0.99 BS) and *rbc*L (0.97 BS). The strain HSB02, identified as *Amphora berolinensis* appears to be rather isolated within the *Amphora* tree, except for an affiliation with the unidentified strain C10 (INSDC accession number FJ002132) in the *rbc*L tree (0.89 BS; Fig. 7b). All strains identified as *Amphora pediculus* cluster in one clade in 18S V4 (0.90 BS; Fig. 7a) and *rbc*L (Fig. 7b). This includes also the strain D54_002 named *Amphora* sp. aff. *atomoides*. The tree derived from *rbc*L sequences also includes the strain AT-21.206 (INSDC accession number AN502022) identified as *Amphora* cf. *fogediana* (Fig. 7b), which forms a branch with strain s0992 named *Amphora copulata* (INSDC accession number AB754831) in 18S V4 adjacent to the *Amphora pediculus* clade (Fig. 7a). In respect to the other strains available from the INSDC databases there is no topology consistent with the taxonomic identifications found in the trees (Fig. 7a, 7b). Several taxa, including the species *Amphora coffeaeformis*, *Amphora normannii* and *Amphora montana* were recently transferred to the genus *Halamphora* [37]; these taxa and also the two INSDC accessions listed as *Halamphora* in the (numbers AB754832, AB754833;) are forming a loose cluster in the upper part of the 18S V4 tree (Fig. 7a). The *rbc*L data set supports an independent clade for the taxa of the genus *Halamphora* (*Amphora coffeaeformis*, *Amphora normannii, Amphora montana*; 0.97 BS; Fig. 7b). However, within the *Halamphora* clade the strains identified as *Amphora coffaeaformis* are not monophyletic (Fig. 7b).

The trees for the genus *Mayamaea* including all available data from INSDC databases are given in Fig. 7c (18S V4) and Fig. 7d (*rbc*L). All strains identified as *Mayamaea terrestris* are forming an independent clade in both trees (0.84 BS in 18S V4, 1.00 BS in *rbc*L; Fig. 7c, 7d). The strains D06_106 and D06_107 representing *Mayamaea permitis* (Syn.: *Mayamaea atomus* var. *permitis*) cluster together in one clade (in *rbc*L 1.00 BS), however other strains named either *Mayamaea atomus*, *Mayamaea permitis* or *Mayamaea atomus* var. *permitis* show no clear pattern according to their names provided in the INSDC databases (Fig. 7c, 7d).

**Figure 8:**
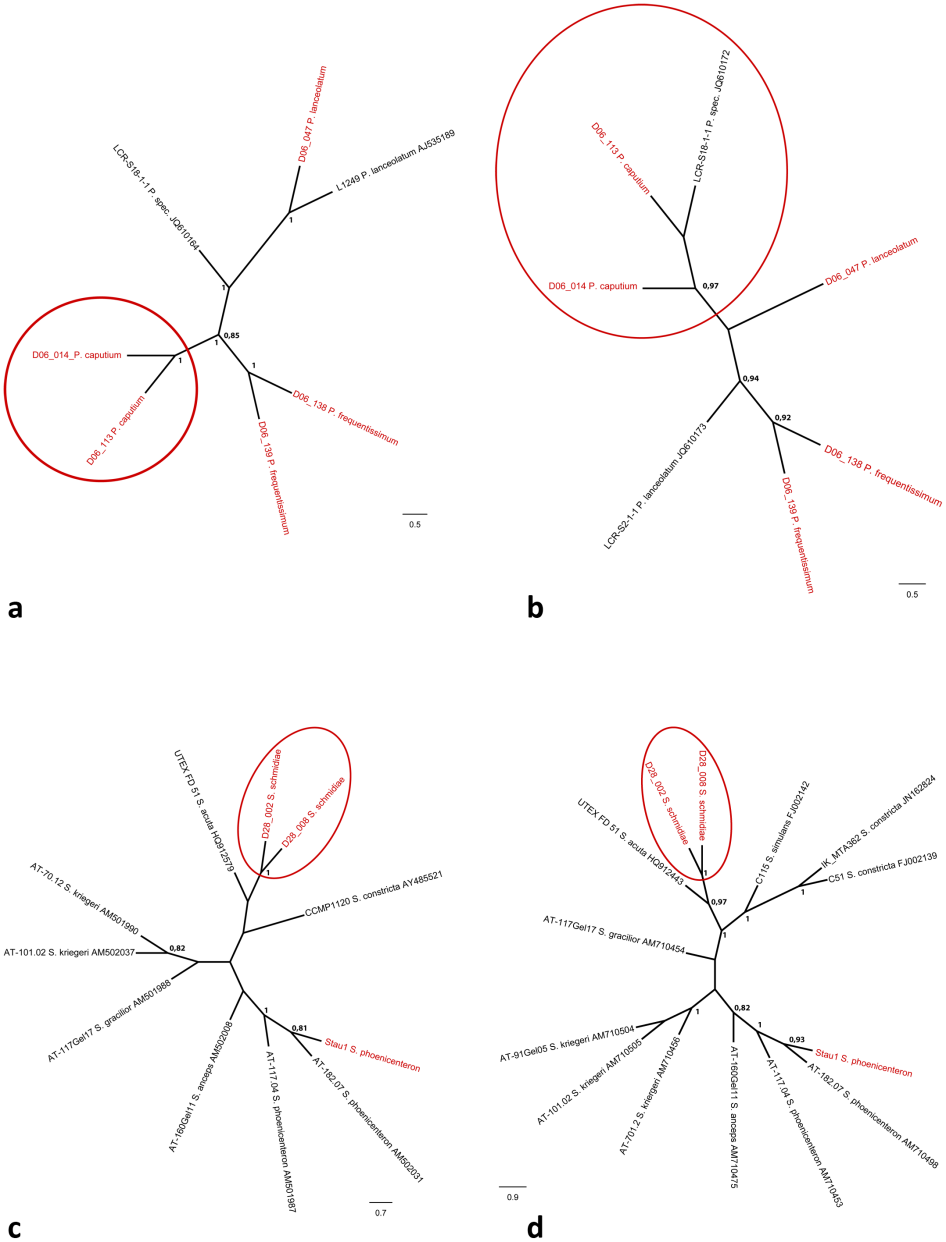
Neighbour Joining Tree (10 000 bootstrap replicates) including all sequences available from the INSDC databases for genus *Planothidium* (a) 18S V4, (b) *rbc*L as well as *Stauroneis* (c) 18S V4, (d) *rbc*L. Bootstrap support values >0.75 given at nodes. Red indicates data from new species.

The trees for the genus *Planothidium* including all available data from INSDC databases are given in Fig. 8a (18S V4) and Fig. 8b (*rbc*L). 18S V4 supports three independent clades for the three including *Planothidium* taxa; namely *Planothidium caputium*, *Planothidium frequentissimum* and *Planothidium lanceolatum* (each taxon supported by 1.00 BS; Fig. 8a). Strain LCR-S18-1-1 (INSDC accession number JQ610164), listed in the INSDC databases as *Planothidium* sp., sits on another branch (Fig. 8a). The topology derived from *rbc*L sequences gives one clade (0.97 BS) for *Planothidium caputium* and strain LCR-S18-1-1 (INSDC accession number JQ610172) plus a second clade for a monophyletic group *Planothidium frequentissimum* (0.92 BS; Fig. 8b). The strains identified as *Planothidium lanceolatum* do not form an independent clade in the *rbc*L analysis (Fig. 8b).

The trees for the genus *Stauroneis* including all available data from INSDC databases are given in Fig. 8c (18S V4) and Fig. 8d (*rbc*L). The *Stauroneis schmidiae* strains (D28_002, D28_008) cluster in one clade, which is sister to the strain UTEX FD 51 (INSDC accession numbers HQ912579 (18S V4) and HQ912443 (*rbc*L)) in both analyses (1.00 BS; Fig. 8c, 8d). The strain Stau1 is identified as *Stauroneis phoenicenteron* and forms a monophyletic clade (1.00 BS for both markers) with all the other accessions with this name available from the INSDC databases (AT-18.207 (INSDC accession numbers AM502031 (18S V4) and AM710498 (*rbc*L)) and AT-11.704 (INSDC accession numbers AM501987 (18S V4) and AM710453 (*rbc*L))). The other taxa available from the INSDC databases also cluster taxonomically consistent, however there are difference in the overall topology recovered from 18S V4 respectively *rbc*L sequences (Fig. 8c, 8d).

### Nomenclatural and taxonomical consequences

Two new taxa were first discovered by morphological means namely *Amphora berolinensis* and *Stauroneis schmidiae*. The analysis of molecular data suggested the existence of two more previously undetected taxa that could later be also morphologically confirmed (*Mayamaea terrestris*, *Planothidium caputium*). For yet another two taxa morphological data is incomplete (teratological outline, micro-morphological data missing) but the molecular data show that they both are different from an identified taxon in this genus; these strains are named sp. (*Amphora* sp. aff. a*tomoides*); in one case we used the term cf. (*Amphora* cf. *pediculus*) to show that it is closely related to a known taxon.

### *Amphora* cf. *pediculus*

**The strains D03_063 & D03_082** are morphologically very similar to our *Amphora pediculus* D03_074 but have double areolae in each ventral stria and not only a single elongated areola like *A. pediculus*. The specimens of these strains have a similar valve outline as *A. indistincta*, but in SEM the differences are more distinct because in *A. indistincta* the width of the central and dorsal side is almost equal and the striae are composed of elongated areolae.

### *Amphora* sp. aff. *atomoides* Levkov

**The strain D54_002** has a valve semi elliptical with arched dorsal margin, concave ventral margin and narrowly rounded valve ends. Valve length is 10–12.4 µm, breadth 4.6–5 µm. The central area on dorsal side is a rectangular fascia almost extending to the dorsal margin; on the ventral side the much broader fascia is expanding towards the valve margin. Raphe branches linear, filiform. Proximal raphe endings straight, distal raphe endings ventrally deflected. Dorsal striae radiate throughout, 16 in 10 µm.

This species closely resembles *A. atomoides* but differences can be observed in the shape of the central area and valve breadth (7–11 µm in *A. atomoides*). In *A. atomoides* the central area on the dorsal side is small or absent not extending to the valve margin, contrary to our Amphora sp. aff. *atomoides* where the central area presents a rectangular fascia almost extending to the dorsal margin. D54_002 also resembles *A. pediculus* with respect to its valve shape and size. However D54_002 can be differentiated by the valve width (*A. pediculus* is narrower with 2.5–4 µm) the central area (*A pediculus* has a distal raphe dorsally deflected and a central area with a rectangular facia, extended to the dorsal valve margin) and the stria density (*A. pediculus* has more striae 18–24/10 µm). D54_002 can also be differentiated from *A. minutissima* by the shape of valve apices (ventrally bent in *A. minutissima*). Additional observations of more specimens by SEM would be necessary to establish the proper identity of this population from Heiligensee, Berlin.

Four taxa in the genera *Amphora*, *Mayamaea*, *Planothidium*, and *Stauroneis* do not fall within the description of any previously known taxa and are therefore described here as new.

### *Amphora berolinensis* N.Abarca & R.Jahn (Figs. 4.1a–i)

Holotype: B 40 0040871 from strain HSBO2; the holotype is represented by Fig. 4.1d.

Type locality: Germany, Berlin, Heiligensee, N 52.60394°E 13.21499° leg. and isolated by O. Skibbe, August 2011.

*Amphora berolinensis* differs from *A. copulata* (Kützing) Schoeman & Archibald because the latter has bigger valves (19–42 µm length, 5–7.5 µm breadth). In SEM the differences are more distinct. Differences can be observed in the shape of the central area (bordered by striae close to the valve margin in *A. copulata*), the raphe (biarcuate in *A. copulata*) and the morphology of the dorsal striae (crossed by longitudinal bars in *A. copulata*). *A. berolinense* also differs from *A. neglectiformis* Levkov & Edlund by the larger valves of the later (18–53 µm length, 5–7 µm breadth) and the ventral striae which are composed of two areolae in *A. neglictiformis* near the valve ends.

The valves of *Amphora berolinensis* are semi-lanceolate to semi-elliptical, with smoothly arched dorsal margin and straight to slightly concave ventral margin, valve ends rounded. Valve length is 9.5–18.9 µm, breadth 4.7–5.2 µm. Axial area is narrow, slightly arched. The central area on the dorsal side has a rectangular fascia extending to the dorsal margin; on the ventral side the fascia is wider expanding towards the valve margin. Raphe is filiform and more or less straight, in some valves the proximal raphe endings are straight, in others they are dorsally bent and the distal raphe endings are straight and in some valves they are ventrally bent. Dorsal striae are coarsely punctated and radiate throughout, 12–14 in 10 µm. Ventrally striae are radiate, composed of one areola.

*Amphora copulata* (Kütz.) Schoeman & R.E.M. Archibald (concept syn. *Amphora libyca* Ehrenberg, sensu post auct.) is morphologically the closest fit to *Amphora berolinensis*, the latter forms a distinctly different clade according to both 18S V4 and *rbc*L (Fig. 7a, 7b). The sequence difference to strain AT-117.10 belonging to *Amphora libyca* sensu post auct. is e.g. 5 bp for 18S V4 and 35 bp for *rbc*L.

### *Mayamaea terrestris* N.Abarca & R.Jahn sp. nov. (Fig. 4.2a–g)

Holotype: B 40 0040847 from strain D29_009b; the holotype is represented by Fig. 4.2e.

Type locality: Germany, Berlin-Dahlem, agricultural soil, N 52.460833°E 13.296944°, leg. L. Buhr, 21 April 2004, cultures isolated by J. Bansemer.

*Mayamaea terrestris* differs from *Mayamaea atomus* var. *atomus* [47] because the latter is longer and wider and has less striae (8.5–13 µm length, 4–5.5 µm breadth, 19–22/10 µm striae). Also the molecular data differ from *Mayamaea atomus* var. *atomus* entries in the INSDC databases in 5 bp for 18S V4 and 25 bp for *rbc*L of strain AT-115Gel07 and even in 69 bp for *rbc*L of strain AT-199Gel01 (AM710510) [48].

The valves of *Mayamaea terrestris* are narrow linear-elipical, ends obtusely rounded. Valve length is 7–8.7 µm, breadth 3–4.5 µm. Striae are radiate throughout, 22–24 (–26) in 10 µm with c. 50 areolae in 10 µm. Raphe is filiform, the two branches are gently arcuate with distinct central pores. Axial area is slightly broad, widening lanceolately towards the middle of the valve. Central raphe ends expanded by depressions around the central pores and deflected, while the ends of the terminal raphe fissures are deflected to the opposite side.

This new species lives in soil; this is signified by the epithet name.

10 further strains (D27_003 & D27_006 & D27_009 & D28_001 & D28_004b & D28_007b & D29_003b & D30_003 & D30_006b & D30_009) have only low sequence differences for 18S V4 and *rbc*L (Appendix S2) and form a clade clearly different from all the other available *Mayamaea* strains (Fig. 7c, 7d).

### *Planothidium caputium* J.Zimmermann & R.Jahn sp. nov. (Fig. 4.3a–h)

Holotype: B 40 0040871; strain D06_014; the holotype is represented by Fig. 4.3f.

Type locality: Germany, Berlin, small river Wuhle, N 52.52079°E 13.57781°, leg. O. Skibbe, 21 April 2004, cultures isolated by J. Bansemer.

Morphologically, *Planothidium caputium* has a similar outline as *Planothidium lanceolatum* but differs from it by a hood over the depression on the rapheless valve as in *P. frequentissimum*. The difference to *P. frequentissimum* lies in the form and size of the hood; which is bigger, longer and wider in *P. caputium* than in *P. frequentissimum* and the hood has a wider opening; this results in a line-like instead of a horse shoe appearance when focusing through the hood. The uncorrected p-distances show that *Planothidium caputium* sequences differ at least 2.4% (18S V4) respectively 2% (*rbc*L) from *Planothidium frequentissimum*, and 6% (18S V4) respectively 4% (*rbc*L) from *Planothidium lanceolatum* (Appendix S2), this is also represented in the trees including all available *Planothidium* strains (Fig. 8a, 8b).

Valves are elliptical to elliptic-lanceolate, with rounded apices. Valve length is 20–22.9 µm, breadth 5.5–6.4 µm. The striae are radiate on both valves, becoming more radiate towards the apices, with 13–14 in 10 µm. Striae are multiseriate with three to five rows of areolae per stria. The axial area is narrow and linear to lanceolate in both valves. A weak central area on the raphe valve and a horseshoe-shaped collar on one side of the rapheless valve which by focusing in LM another line less arched can be recognized (see also Straub 1990 [49]).

Also strain D06_113 belongs to this species.

### *Stauroneis schmidiae* R.Jahn & N.Abarca sp. nov. (Fig. 4.4a–h)

Holotype: B 40 0040883 from strain D28_008; the holotype is represented by Fig. 4.4f.

Type locality: Germany, Berlin-Dahlem, agricultural soil, N 52.460833°E 13.296944°, leg. L. Buhr, 21 April 2004, cultures isolated by J. Bansemer.

Morphologically, *Stauroneis schmidiae* differs from *Stauroneis borrichii* (Petersen) Lund, which has a similar valve outline but with protracted ends, because the latter is shorter and more slender and has more striae (18–25 µm length, 4.0–5.0 breadth, 20–22 striae and 25–28 punctae per 10 µm (see Van de Vijver et al 2004) and from *Stauroneis pseudomuriella* Van de Vijver & Lange-Bert. (2004) [50] which has similar morphometrics as our new species but more striae (21–42 µm length, 5–6.5 µm breadth, 20–22 striae and 25 punctae per 10 µm) but this species has no pseudosepta.

Valves are linear-lanceolate with very slightly rounded non-protracted ends. Valve length is 27–28.2 µm, breadth 5.5–6 µm. Striae are radiate throughout the entire valve, 15–18 in 10 µm. Puncta of the striae are discernible in LM and are 24–28 in 10 µm. Pseudosepta present.

Also strain D28_002 belongs to this species.

Compared to the other available *Stauroneis* strains *Stauroneis schmidiae* clusters independently for both markers (Fig. 8c, 8d).

This species is named in honor of Prof. Dr. AnnaMaria Schmid who was an inspiring diatom teacher to Regine Jahn.

## Discussion

The 37 naviculoid diatom taxa, of which reference barcodes are published here, represent only about 7% of the total diatom flora which is 14% of the naviculoid taxa recorded for Berlin waters (539 taxa, see [31]). Nevertheless, it is a first milestone in characterising diatoms not only by morphological but also by molecular means, which represents the start of a taxonomic reference library for diatoms.

Identification via DNA sequences is an important tool, especially in microorganisms. Many of the large scale environmental DNA barcoding studies in protists so far rely on higher taxonomic levels of families and above; only rarely they reach a resolution at genus level. In diatoms, assignment to genus level is unproblematic [51], [52]. Even identification to the species level is possible, but strongly depends on the quality of the reference database [52]–[58]. We here tested the taxonomic consistency of naviculoid diatom taxa at the species level by comparing our identified sequences with the published sequences in the repositories of the INSDC. We found that the taxonomic assignment in INSDC is currently unsatisfying, because it is often erroneous. In the data of the two commonly used DNA barcoding markers for diatoms 18S V4 and *rbc*L we analysed, we found that for *rbc*L 26% for the sequences listed under the same name as our strains more than 15 bp sequence difference were recorded (Fig. 5b); for 18S V4 this was 12% (Fig. 5a). For the 800 bp long *rbc*L fragment 15 bp difference amounts to roughly 2% sequence difference, in the shorter (400 bp) 18S V4 fragment 15 bp difference correlates to even 4%. The relatively high percentage of differences in these short DNA fragments suggests that the sequences belong to a different taxon. This implies morphology-related misidentification, mislabelling or cross-contamination. There are an additional 16% (*rbc*L, Fig. 5b) respectively 5% (18S V4, Fig. 5a) of the sequences where sequences with the same taxon name showed differences between 6 and 15 bp, here it is unclear whether these strains belong to a different taxon of a closely related cryptic species or whether they reflect natural intraspecific variation. Furthermore, we found that in 4% (*rbc*L, Fig. 5b) respectively 15% (18S V4, Fig. 5a) of the cases, identical sequences in the repositories of the INSDC were annotated with a different taxon name than the strains of this study. These sequences therefore provide an erroneous identification. In summary, the unevaluated use of information deposited in the INSDC leads to wrong identifications in at least 30% of the cases; in only about 20% of our cases, the identifications coincided unambiguously.

Unfortunately, in most cases it is not possible to trace the DNA sequence to the specimen from which it originated and, because of lacking voucher specimens, taxonomic evaluation is not possible; hence there are no means to verify whether a faulty taxon assignment had occurred or an interesting biological phenomenon. Therefore such sequences are of no future use and valuable information is lost to science. Assessment of diatom community composition through environmental DNA barcoding could greatly benefit from better documented reference libraries, especially because biodiversity in general should be evaluated at least on the species level [59].

Furthermore, the linkage between historically and morphologically described taxa and molecular sequences is not very strong. A possible threat is that two independent data clouds might develop [60]: one including large amounts of molecular data from environmental sequencing, the other species specific data (e.g. paleontological and recent distribution, ecology, phylogeny) linked to morphological descriptions. For organism groups where next to no morphology based data exist (e.g. many groups of bacteria), there is little harm if the information in the two clouds cannot be correlated. However, in groups like diatoms, where two centuries of data collection linked to morphologically described species exists, it would be a waste of painfully acquired data not to link these two groups of data. At the moment, this link would be a reference sequence that is connected to a morphological voucher (and DNA sample) deposited in a natural history collection and therefore available for multiple testing and verification of results as well as for long-term studies.

We here define a taxonomic reference library as an entity combining molecular data – in our case DNA sequence data of two markers – with morphological documentation of important features as well as a valid name. Also environmental information on the collecting site should be provided in a standardised format.

Documentation should also include the deposition of DNA in a curated repository. To ensure traceability of a name/sequence back to the specimen it originated from, morphological details important for identification should be provided in an online photographic documentation, this includes high-resolution photographs giving an overview of the cell as well as details produced by electron microscopy or comparable techniques. Another special aspect for diatoms (and some other microorganism groups) is that many sequences derive from cultured clonal strains, especially if they are linked to morphological entities. Therefore, the strain number and other strain specifications are valuable information that should be presented along with the sequence.

Ideally, all the necessary information for traceable taxonomic classification should be available in a single data portal; however, at the moment there are several technological limitations to deposit and/or respectively retrieve all the information in and from one location. The Consortium for the Barcode of Life (CBOL) aims at compiling DNA barcode records in a public library (Barcoding of Life Database BOLD) [53] and even designed a Barcode Submission Tool for submitting sequences to the INSDC databases. However, this tool is limited to one marker, namely the mitochondrial cytochrome oxidase subunit I (COI) e.g. [17], [61]–[65]. For many groups, e.g. plants [66] but also diatoms, this barcoding marker is not routinely applicable [19], [21]–[25], albeit there are BOLD supported activities to implement alternative solutions for some organism groups e.g. [28]. On the other hand, the Barcode Submission Tool provides possibilities to at least upload a pherogram (output of sanger sequencing), but no pictures of the organisms can be stored. Therefore, this tool does not require a link to a morphological voucher (digital and physical), which would allow for subsequent taxonomic validation. Also a link to a herbarium specimen is only indirectly possible if the accession number of the specimen collection is given and the respective collection has their specimen picture online available. Although, it seems generally possible to deposit pictures and other data along with the DNA sequence in BOLD [53], unfortunately, the data deposited within BOLD is often not open access, depending on the rights given by the administrator. Also, we heard reports that data is not released to the public even if requested by the author. In conclusion it would be preferable if INSDC would extend their service, as they are the most commonly used platform to deposit sequence data [58].

Here we present our strategy on how documentation can be performed to build a comprehensive reference database for diatoms even with inconvenient IT possibilities. The here presented materials and data have been documented as follows: The physical vouchers (microscopic slides and SEM stubs) have been deposited in the Berlin Herbarium (B), the DNA in the DNA bank network of the Botanic Garden and Botanical Museum Berlin-Dahlem [39]. The data for both items are made available through The Global Genome Biodiversity Network (GGBN [67]) and The Global Biodiversity Information Facility (GBIF [68]). The sequences have been submitted to an INSDC database (EMBL) along with strain numbers, voucher number from the Berlin Herbarium (B) and DNA bank number. Also primer details and geo-references have been deposited there. Photographic documentation is online available from the AlgaTerra Information System [30], linked through INSDC accession number and accession number from the Berlin Herbarium. Morphological characters, cultivation details as well as sampling data of the collecting sites beyond the geo-references (e.g. ecological specifications) have also been deposited in the AlgaTerra Information System [30].

A carefully documented reference sequence could be considered as something similar to a molecular type of the name of a species. Biological taxon types should be documented with a maximum amount of data, which makes it possible for every researcher to determine whether a specific specimen belongs to the concept of the designated type. In the botanical [69] and zoological [70] codes of nomenclature the basis for species description is the deposition of physical specimen. A reference sequence or reference barcode should be similarly well documented.

Biological identification systems are in constant development, therefore a continuous process of confirmation, validation and updating in relation to alpha taxonomy is required to build a compressive and accurate reference library. Protocols for data curation and revision are indispensable for new species discovery as well as taxonomic revisions. Therefore, entries in a taxonomic reference library (e.g. in an extended INSDC like system) need to be curated and updated in order to be in line with current taxonomy. However, a huge impediment for data curation by the respective author – once it is submitted – is, that there is no reward system for researchers for curating their data [71]. It has been shown, that incentives for researchers for the publication of thoroughly documented datasets similar to the publication of the conclusions drawn from these could greatly increase the motivation to publish datasets [71]. Another approach would be that data curation would be carried out by professional personnel employed for this purpose or a combination of both approaches.

Not only DNA barcoding approaches would benefit from well documented and referenced molecular data but also taxonomic and phylogenetic studies of diatoms which could integrate published data more efficiently if better documentation linked to physical objects were available [72]. For example, the clusters found for the genus *Mayamaea*, based on available 18S V4 and *rbc*L sequences, show low taxonomic consistency (Fig. 7c, 7d). The INSDC data suggest that there are different groups of *Mayamaea* (*atomus* var.) *permitis*, and within the *Mayamaea atomus* (var. *atomus*) sequences is one sequence named *Mayamaea fossalis* var. *fossalis* (Fig. 7c, 7d, black and red). For two of the AT strains included in the *Mayamaea* analysis additional data is available from the AlgaTerra Information System [30] (Fig. 7c, 7d, green): (a) more taxonomic detail is given than deposited alongside the sequence in INSDC - strain AT-115Gel07 is identified as *Mayamaea atomus* var. *atomus* and AT-101Gel04 as *Mayamaea atomus* var. *permitis* - and (b) photographs with morphological details are provided. Therefore the identification of both strains could be checked and verified. Even though additional data for only two strains is available from the AlgaTerra Information System [30], this already aids in the interpretation of the trees given in Fig. 7c and 7d; especially for the tree based on 18S V4. There is a cluster of *Mayamaea permitis* (Syn. *Mayamaea atomus* var. *permitis*), incl. strain AT-101Gel04, and one strain (AT-115Gel07) belonging to *Mayamaea atomus* var. *atomus* (Fig. 7c, green). As *Mayamaea permitis* (Syn. *Mayamaea atomus* var. *permitis*) has been raised to species rank due to morphological reasons (see above), this allows the interpretation that *Mayamaea fossalis* could be an independent taxon (Fig. 7c, green). For the tree based on *rbc*L, however, only an informed guess can be made: for two strains, namely (Wes2)f and AT-199Gel01, no additional data is available to check the identification (Fig. 7d). If it could be assumed that (Wes2)f was misidentified and AT-199Gel01 belongs to *Mayamaea permitis*, again four independent taxa could be assumed: *Mayamaea atomus*, *Mayamaea fossalis*, *Mayamaea permitis* and *Mayamaea terrestris*. This example, particularly the different interpretation possibilities between 18S V4 and *rbc*L trees, clearly shows how valuable additional data can be for the interpretation of sequence based analyses.

Due to the fact that species descriptions in diatoms are based on morphology derived from microscopic pictures (of variable quality) of single, or a limited number, of valves from a presumed population in mixed samples, it is often difficult to unambiguously identify a strain. Even within a single clonal culture, morphological variation sometimes fits in parts to different species circumscriptions [45]. In addition, size wise clonal cultures are often at the lower end of the morphometrics of a taxon description; if cultured for too long and if no auxosporulation has taken place, diatom valves tend to lose their typical morphological features because they get smaller with each cell division. This leads to the problem how to link sequences derived from cultures to a type specimen or at least to a current species concept. If a type specimen is designated, this can be achieved e.g. through epitypification as has been done for *Cocconeis pediculus* and *C. placentula* [73], [74]. But in most cases, this will be done in the context of a taxonomic revision of a species group as e.g. for *Gomphonema saprophilum* [45] and needs to be done for the two unidentified *Pinnularia* species of this study. For the purpose of a reference library, if no unambiguous identification seems possible, the sequence could either be designated as belonging to a certain “formenkreis” (taxon group) marked as *affine* (e.g. *Amphora* sp. aff. *atomoides*), as not exactly fitting the original descriptions marked as *confer* (e.g. *Amphora* cf. *pediculus*) [http://bionomenclature-glossary.gbif.org/], or a new taxon has to be described formally along with providing the reference sequence (e.g. *Amphora berolinense*). The first two options are a practical way to make re-users of the data aware of an “uncertainty level” concerning the taxonomic identification; this is better than providing no guidance to the species group by giving just the genus name such as *Amphora* sp. As we documented in this study, the marine or halophilic species of *Amphora* sensu lato have been recently moved into the genus *Halamphora*; for a freshwater reference library, this is important ecological data. In addition, this information might become valuable for the interpretation of taxonomic discrepancies

## Conclusions

As here shown exemplarily for some naviculoid diatoms, taxonomic reference libraries could serve as an online accessible and algorithmically searchable equivalent to commonly used printed identification literature. They are needed to link molecular based identification technologies with correct organism references. However, up to now searchable data bases often include large percentages of wrongly annotated sequencesand provide no possibility to trace the identification back to the respective specimen, leaving molecular based techniques often with identifications only to family or genus level. While for some studies this level of taxonomic depth seems to suffice (e.g. large scale biodiversity assessments), there are many studies that could profit from well documented molecular data (e.g. species inventories, monitoring, taxonomy, phylogeny). Therefore, it would be worth the effort to provide all material needed for identification of an organism.

## Supporting Information

Appendix S1 **Table of INSDC accessions with strain numbers and references.**
(XLSX)

Appendix S2 **Table with uncorrected p distances given for individual genera.** Intra-taxon variability is highlighted.
(XLSX)

Appendix S3 **Neighbour Joining Trees (10 000 bootstrap replicates) derived from individual datasets 18S V4 including all sequences from this study.** All bootstrap support values given above branches.
(TIFF)

Appendix S4 **Neighbour Joining Trees (10 000 bootstrap replicates) derived from individual datasets** ***rbc*** **L including all sequences from this study.** All bootstrap support values given above branches.
(TIFF)
