# Correction: Taxonomic Reference Libraries for Environmental Barcoding: A Best Practice Example from Diatom Research

**DOI:** 10.1371/journal.pone.0114758

**Published:** 2014-11-26

**Authors:** The PLOS ONE Staff

The third author’s name is spelled incorrectly. The correct spelling is: Neela Enke. The correct citation is: Zimmermann J, Abarca N, Enke N, Skibbe O, Kusber W-H, et al. (2014) Taxonomic Reference Libraries for Environmental Barcoding: A Best Practice Example from Diatom Research. PLoS ONE 9(9): e108793. doi:10.1371/journal.pone.0108793.

In the Results section, the heading “*Amphora berolinensis* N.Abarca & R.Jahn (Figs. 4.1a–i)” should read “*Amphora berolinensis* N.Abarca & R.Jahn sp. nov. (Figs. 4.1a–i)”. In the third paragraph under this heading, “*A. berolinense*” should appear as “*A. berolinensis*”.

In the paragraph directly above the Conclusions, “*Amphora berolinense*” should appear as “*Amphora berolinensis*”
