# Supplementary figures and images for: Taxonomic Reference Libraries for Environmental Barcoding: A Best Practice Example from Diatom Research
Source: PLoS One. 2014 Sep 29;9(9):e108793. doi: 10.1371/journal.pone.0108793 (PMC4180937; doi:10.1371/journal.pone.0108793)

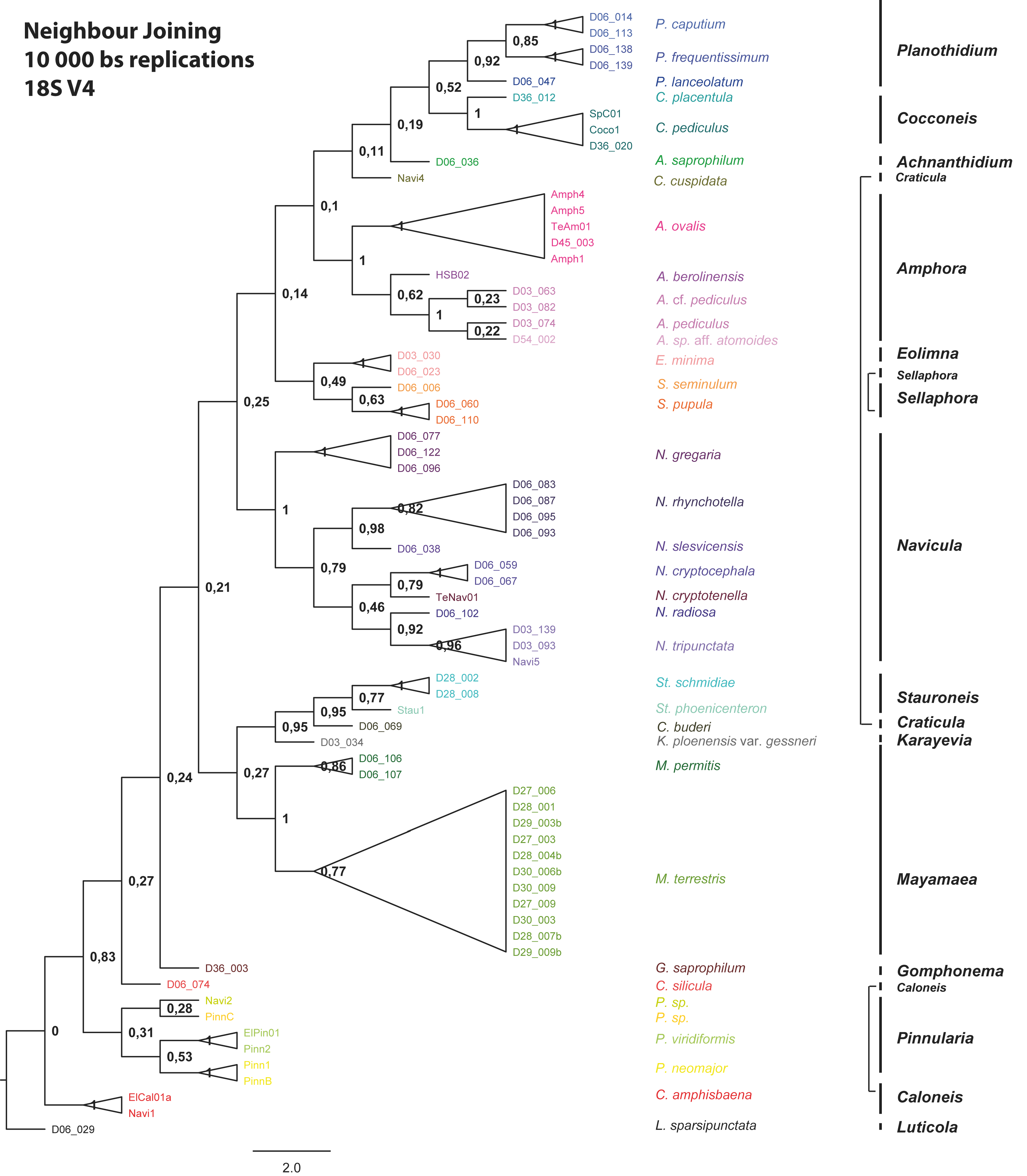

Supplement: Appendix S3 — Neighbour Joining Trees (10 000 bootstrap replicates) derived from individual datasets 18S V4 including all sequences from this study. All bootstrap support values given above branches. (TIFF) [file pone.0108793.s003.tiff]

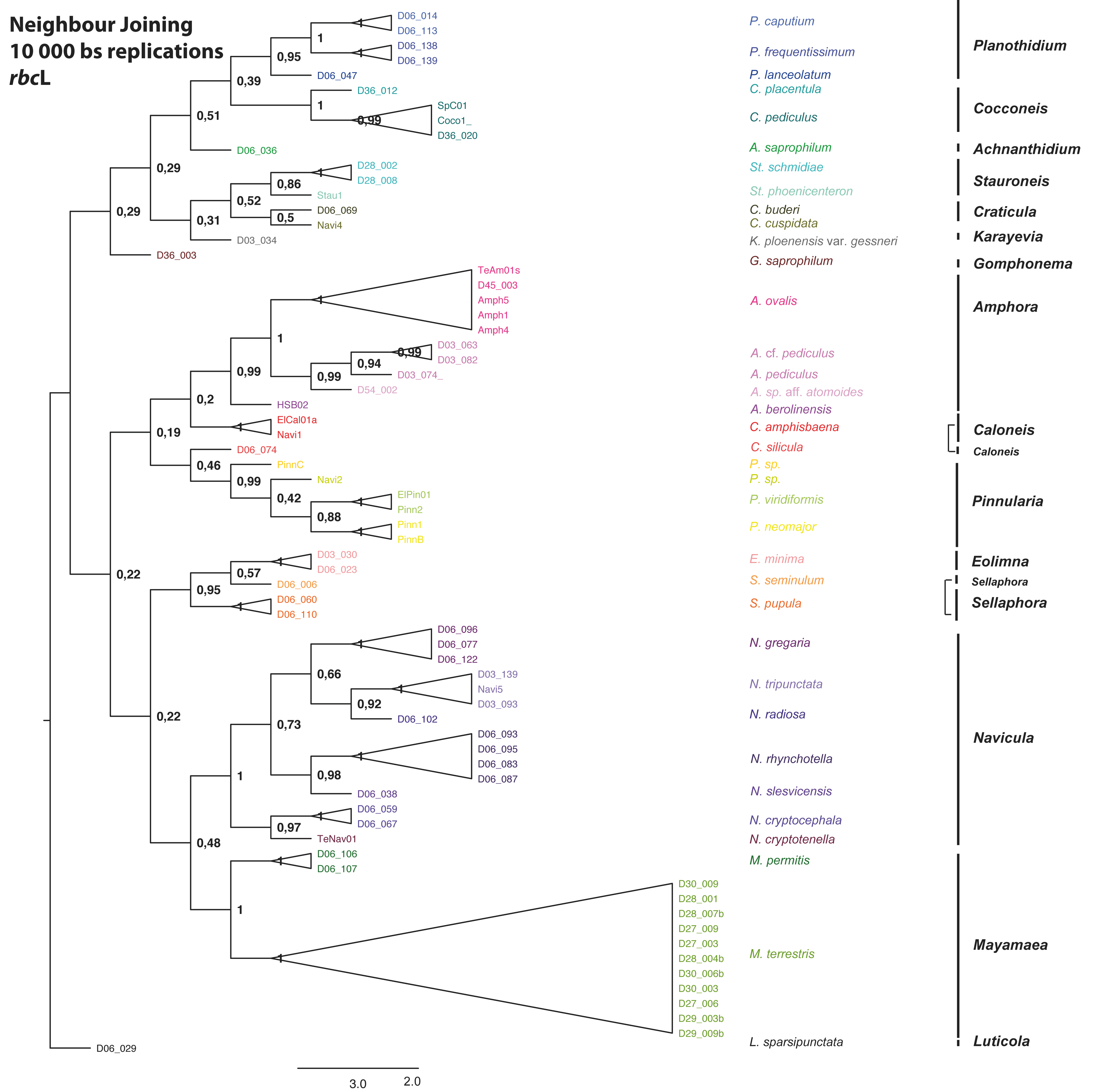

Supplement: Appendix S4 — Neighbour Joining Trees (10 000 bootstrap replicates) derived from individual datasets rbc L including all sequences from this study. All bootstrap support values given above branches. (TIFF) [file pone.0108793.s004.tiff]
